# Development and Characterization of a Multiplex Assay to Quantify Complement-Fixing Antibodies against Dengue Virus

**DOI:** 10.3390/ijms222112004

**Published:** 2021-11-05

**Authors:** Eduardo J. M. Nascimento, Brooke Norwood, Allan Parker, Ralph Braun, Eloi Kpamegan, Hansi J. Dean

**Affiliations:** Takeda Vaccines, Inc., 40 Landsdowne Street, Cambridge, MA 02139, USA; Brooke.Norwood@Takeda.com (B.N.); Allan.Parker@Takeda.com (A.P.); Ralph.Braun@Takeda.com (R.B.); Eloi.Kpamegan@Takeda.com (E.K.); Hansi.Dean@Takeda.com (H.J.D.)

**Keywords:** Luminex, dengue virus, C1q, complement system, complement-fixing antibodies, classical pathway

## Abstract

Antibodies capable of activating the complement system (CS) when bound with antigen are referred to as “complement-fixing antibodies” and are involved in protection against *Flaviviruses*. A complement-fixing antibody test has been used in the past to measure the ability of dengue virus (DENV)-specific serum antibodies to activate the CS. As originally developed, the test is time-consuming, cumbersome, and has limited sensitivity for DENV diagnosis. Here, we developed and characterized a novel multiplex anti-DENV complement-fixing assay based on the Luminex platform to quantitate serum antibodies against all four serotypes (DENV1-4) that activate the CS based on their ability to fix the complement component 1q (C1q). The assay demonstrated good reproducibility and showed equivalent performance to a DENV microneutralization assay that has been used to determine DENV serostatus. In non-human primates, antibodies produced in response to primary DENV1-4 infection induced C1q fixation on homologous and heterologous serotypes. Inter-serotype cross-reactivity was associated with homology of the envelope protein. Interestingly, the antibodies produced following vaccination against Zika virus fixed C1q on DENV. The anti-DENV complement fixing antibody assay represents an alternative approach to determine the quality of functional antibodies produced following DENV natural infection or vaccination and a biomarker for dengue serostatus, while providing insights about immunological cross-reactivity among different *Flaviviruses*.

## 1. Introduction

Dengue virus (DENV) is a *Flavivirus* with four different but antigenically related serotypes (DENV1-4) that cause a wide range of symptoms from dengue fever, a debilitating flu-like disease, to the more severe and sometimes lethal manifestation of dengue hemorrhagic fever. DENV affects tropical and subtropical areas of the world where the mosquito vector (mostly *Aedes aegypti*) is present. DENV exposure induces production of IgM, IgG, and IgA immunoglobulins that target pre-membrane (prM), envelope (Env), and other viral proteins, resulting in virus neutralization through inhibition of receptor-mediated entry and/or host membrane fusion [1,2,3]. Interestingly, the potency of neutralizing antibodies against DENV and other *Flaviviruses* (e.g., West Nile virus) is enhanced in the presence of proteins of the complement system (CS) [4,5,6], suggesting that the repertoire of antibody functions against DENV is more complex than the antibody–viral interactions measured in standard neutralizing antibody assays. 

The CS is an arm of the innate immune system that is one of the effector mechanisms of antibody responses [7]. Although there are three distinct pathways through which the CS can be activated, the classical pathway (CP) is initiated by antibody–antigen complexes or pattern recognition molecules (e.g., complement component 1q (C1q)) present in pathogen surfaces [8,9]. C1 complex, the first component of the CP, is composed of the subcomponents C1q, C1r, and C1s, and recognizes the Fc region of the antibody–antigen complex via C-terminal globular head regions of C1q [8]. Fixation of C1q onto antigen–antibody immune complexes or pathogen surfaces initiates a cascade of events including proteolytic activation mediating downstream effects leading to deposition of various CS proteins onto virus particles.

Antibodies capable of activating the CS when bound with antigen are referred to as “complement-fixing antibodies”, and mainly involve the IgM class (or isotype), as well as subclasses 1 and 3 within the IgG isotype. In the past, complement-fixing antibody assays were used as a diagnostic tool for many *Arbovirus* infections, including DENV [10,11,12,13]. These assays require critical reagents that include viral antigens prepared from infected mouse brain extracts, sheep red blood cells, and unpurified guinea pig serum which are difficult to standardize [14]. The test is time-consuming and cumbersome requiring long incubation times (up to 20 h) with lower performance relative to neutralization and hemagglutination inhibition tests [13,15]. Until today, no improved method for detecting DENV-reactive, complement-fixing antibodies has been developed.

The ability of antibodies to activate the CS has recently been associated with anti-viral protective immunity and in the development of humoral immune response. Formation of the membrane attack complex (C5b-9) triggered by antibody–antigen complex inactivates enveloped viruses while deposition of C3b facilitates pathogen phagocytosis [15,16]. Additionally, C3d deposition improves antigen retention in secondary lymph organs, B-cell clone selection, and antibody affinity maturation, while enhancing antibody production [7,17]. Thus, it is important to develop a sensitive and specific method to evaluate the ability of antigen-specific antibodies to activate the CS.

Here we describe the characterization of a novel Luminex-based multiplex assay to quantify serum antibodies capable of fixing C1q and activating the CS when bound to prM/Env proteins of all DENV serotypes expressed on virus-like particles (VLPs). The complement-fixing antibody assay demonstrated good reproducibility and linearity, as well as high sensitivity and specificity relative to a microneutralization assay. In non-human primates, antibodies produced in response to primary DENV1-4 infection induced CS activation against homologous and heterologous serotypes. In addition, antibodies produced following vaccination against Zika virus (ZIKV) but not other flavivirus vaccines fixed the CS on DENV structural proteins. The anti-DENV complement-fixing antibody assay represents an alternative approach to determine the quality of antibodies produced following DENV natural infection or vaccination and is a possible biomarker for dengue serostatus, while providing insights about serologic relationships among different *Flaviviruses*. The assay format also has potential application to studying complement-fixing antibodies against other pathogens.

## 2. Results

### 2.1. Characteristics of the Anti-DENV Complement Fixing Antibody Assay

The characterization of the anti-DENV complement-fixing antibody assay, based on C1q fixation by DENV-specific antibodies, was performed as described in Section 4. The assay’s limit of detection (LOD) and limit of quantitation (LLOQ) were estimated and shown in Supplemental Tables S1–S6. The assay LLOQ for all DENV serotypes was 3 EU/mL on average (ranging from 2 (DENV4) to 4 (DENV1) EU/mL; Supplemental Figures S1 and S2 and Supplemental Tables S2–S6). The assay had low background signal (as determined using a titration curve of the negative control serum; Supplemental Figure S1) and had good linearity, where the observed and expected complement-fixing antibody concentrations of the assay reference at various dilutions linearly correlated, with slopes and 95% confidence intervals close to 1 (Supplemental Figure S3). Overall, complement-fixing antibody concentrations were comparable, independent of the run, the control sample used, the DENV VLP, or the operator (Supplemental Figures S4–S6). Coefficient of variance for intra (same operator) and inter-experiment precision (multiple operators and days) were consistently below 20% and 21%, respectively, for all assay control samples and DENV VLPs evaluated (Supplemental Figures S5 and S6). 

**Figure 1:**
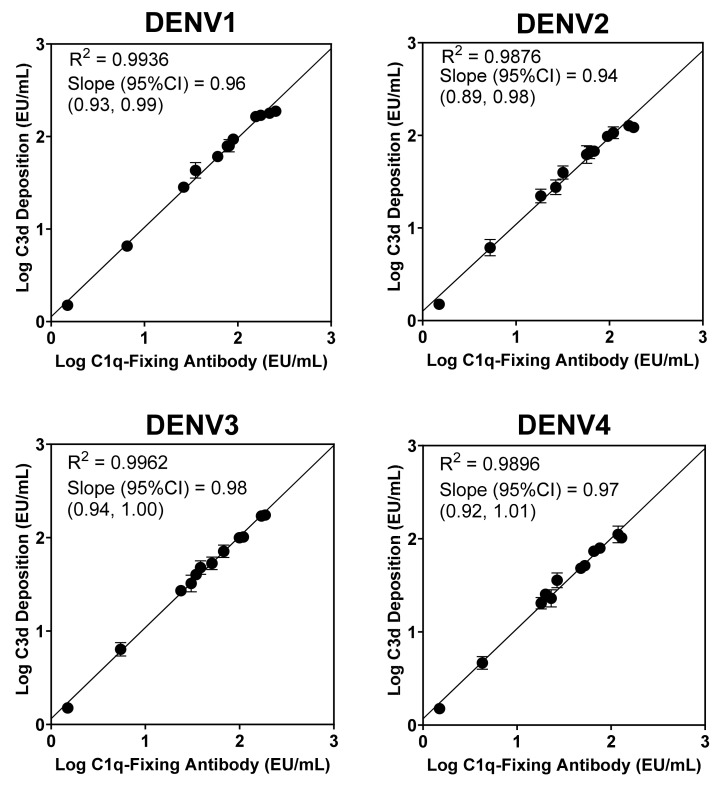
Correlation analysis between complement-fixing antibodies based on C1q fixation and C3d deposition mediated by dengue virus-specific antibodies. Correlation analysis was performed using Log10-transformed C1q-fixation and C3d-deposition antibody concentrations. Correlation coefficient (R^2^) and slopes with a 95% confidence interval were calculated for each DENV serotype.

Initially, we determined if the anti-DENV complement-fixing antibody assay based on fixation of C1q translates to complement C3d deposition, an early marker of complement system activation known to be involved in increased B cell responses [17,18]. A panel of 12 samples from healthy subjects who were seropositive for DENV, with a wide range of complement-fixing antibody levels (Supplemental Figure S7A), was used to measure C3d deposition by DENV-specific antibodies on three independent occasions using a C3d deposition Luminex assay (see Section 4). The pattern of C3d deposition levels was similar to complement-fixing antibody measured (Supplemental Figure S7B). Both biomarkers were highly correlated, with a correlation coefficient (R^2^) and slope close to 1 irrespective of the DENV serotype (Figure 1), suggesting that C1q fixation measured by the assay could be used as a surrogate marker for CS activation by antigen-specific antibodies.

### 2.2. Anti-DENV Complement-Fixing Antibody Luminex Assay Comparisons to a Dengue Microneutralization and Dengue Total IgG Binding Assay

**Figure 2:**
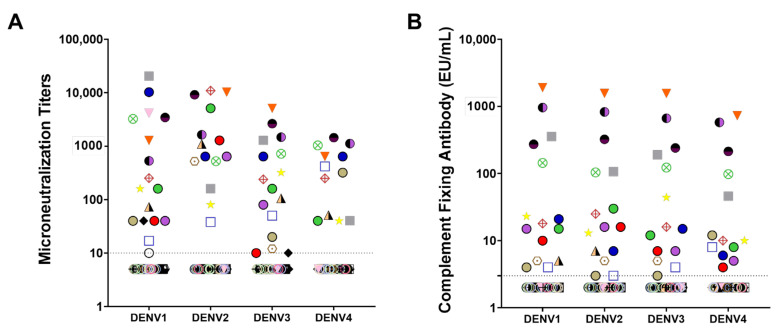
Neutralizing and complement-fixing antibody levels against all four dengue virus serotypes in subjects living in dengue endemic areas. Microneutralization (MNT50) (**A**) [17,18] and complement-fixing (**B**) antibody levels were determined from subjects participating in phase II clinical trials DEN-203 and DEN-204 before vaccination with tetravalent live-attenuated dengue vaccine TAK-003. Horizontal lines represent the threshold (>10 for MNT50 and >3 EU/mL for complement-fixing antibodies) from which dengue serostatus was determined. Each study participant is represented by a unique symbol/color combination.

**Figure 3:**
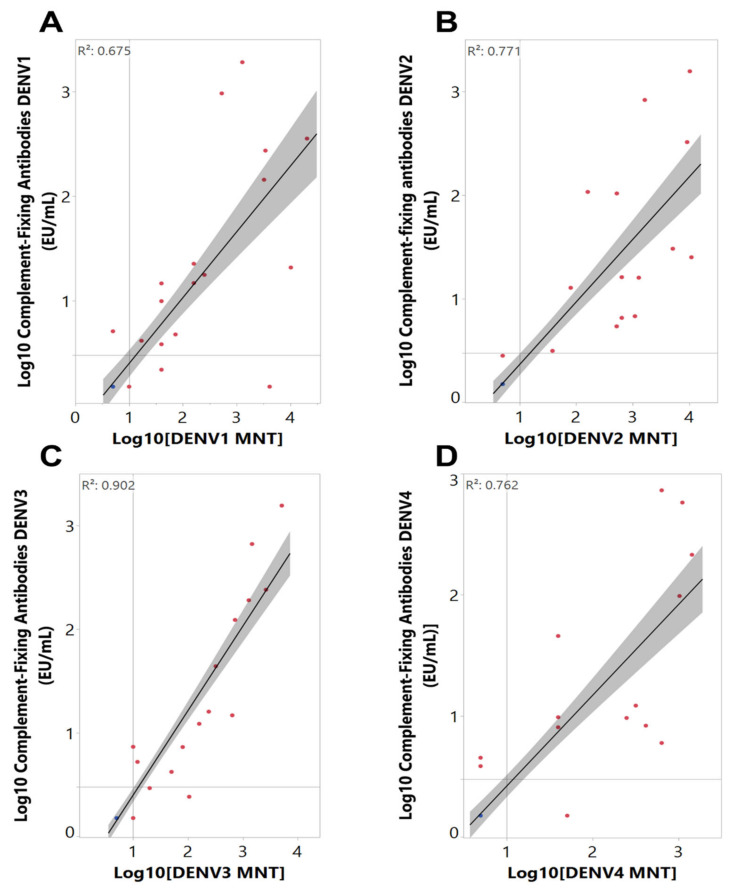
Correlation analysis between neutralizing (MNT50) and complement-fixing antibodies against dengue virus (DENV) serotypes 1 (**A**), 2 (**B**), 3 (**C**) and 4 (**D**) in subjects living in dengue endemic areas. Correlation analysis was performed using Log10-transformed MNT50 and complement-fixing antibody levels and the correlation coefficient (R^2^) was calculated for each DENV serotype. Samples are color coded by DENV serostatus determined by MNT50. Blue symbols represent seronegative study participants, and red represents seropositive ones. Vertical and horizontal lines represent MNT50 titer of 10 and complement-fixing antibody concentration of 3 EU/mL. The shaded gray represents the confidence region for the fitted line.

A panel of 53 samples collected from children and adults who participated in clinical trials (Table 1), either seronegative (*n* = 35 or 66%) or seropositive (*n* = 18 or 34%) to DENV at baseline as determined by a validated dengue microneutralization assay (MNT_50_) [19,20], was used to assess the performance of the anti-DENV complement-fixing antibody assay to determine dengue serostatus following natural virus exposure. Figure 2 and Table 2 depict the overall distribution and geometric mean antibody titers, respectively, of each sample against all DENV serotypes by both assays. Geometric mean MNT_50_ titers ranged from 12 (DENV4) to 20 (DENV2; Table 2) and the complement-fixing antibody geometric mean concentrations ranged from 4 (DENV4) to 5 EU/mL (DENV1; Table 2). When the relationship between MNT_50_ and complement-fixing antibodies was investigated, moderate (R^2^ = 0.675 for DENV1) to high (R^2^ = 0.902 for DENV3) correlations were observed (Figure 3). Moreover, virus-specific total binding IgG concentration was determined in the same sample panel (Supplemental Figure S8) and was also found to correlate with complement-fixing antibody levels (Supplemental Figure S9).

### 2.3. Sensitivity and Specificity of the Complement-Fixing Antibody Assay Compared with the Microneutralization Assay for Each Dengue Virus (DENV) Serotype

**Figure 4:**
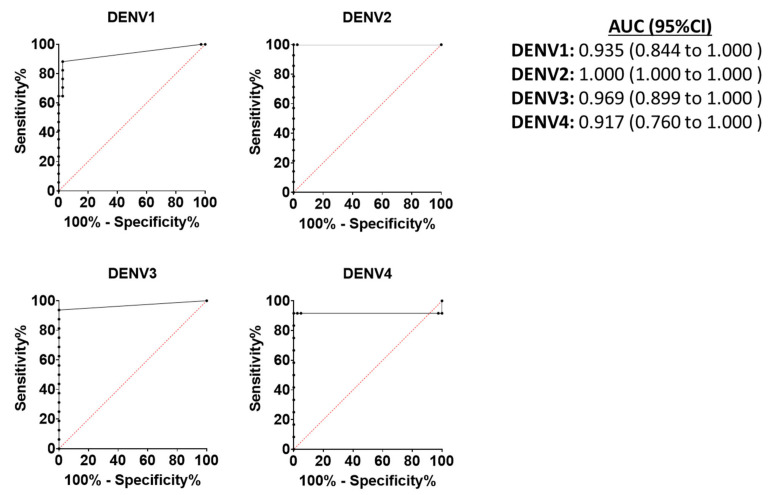
Receiver operating characteristic (ROC) curve analysis to evaluate performance of the complement-fixing antibody assay to determine dengue virus (DENV) serostatus relative to a microneutralization assay (MNT50). Samples from baseline seropositive and seronegative subjects based on MNT50 titers >10 and <10, respectively, were used to evaluate the performance of the complement-fixing antibody assay to evaluate DENV serostatus. Area under the curve (AUC) and a 95% confidence interval (CI) were determined for all four DENV serotypes.

Using MNT_50_ as the gold standard and the threshold complement-fixing antibody concentration of 3 EU/mL, sensitivity of the assay ranged from 82 to 100% depending on the serotype analyzed (Table 3). Assay specificity, on the other hand, ranged from 95% (DENV4) to 100% (DENV2 and DENV3; Table 3). Receiver operating characteristic (ROC) curve analysis [21] indicated that the complement-fixing antibody assay was accurate in determining dengue serostatus relative to MNT_50_ with the area under the curve (AUC) ranging from 0.917 (DENV4) to 1.000 (DENV2; Figure 4). 

### 2.4. Antibodies Produced after Monospecific DENV Infection Fix Complement against Homologous and Heterologous DENV Serotype Antigens

**Figure 5:**
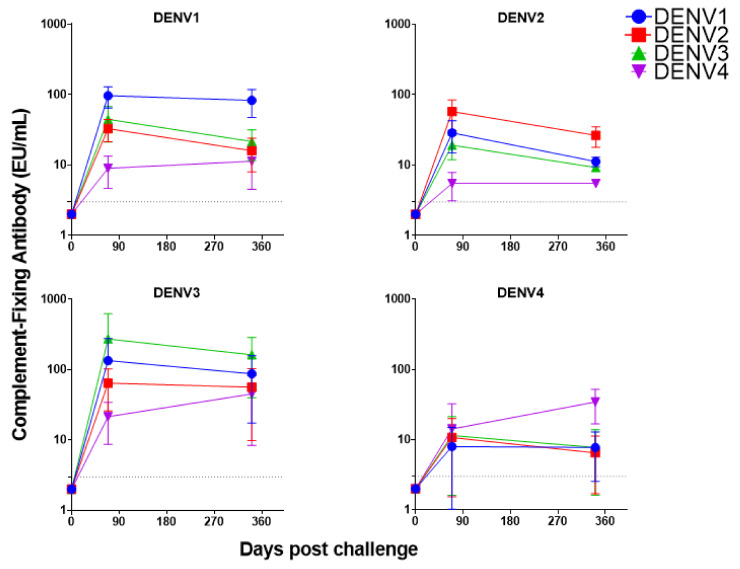
Complement-fixing antibody responses in non-human primates infected with each dengue virus (DENV) serotype. Infecting virus serotype is shown on top of each kinetic curve. Complement-fixing antibody kinetic curves (at days 0, 69, and 341) against DENV1, DENV2, DENV3, and DENV4 are shown in blue, red, green, and purple, respectively. Each point represents the mean and standard deviation of 4 (days 0 and 69) or 3 (day 341) animals. Concentration of 3 EU/mL is shown as dotted line in the y-axis for reference.

**Figure 6:**
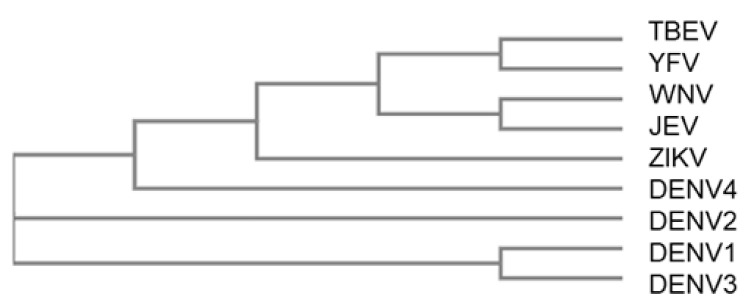
Sequence alignment of the envelope amino acid sequence of different *Flaviviruses* used in non-human primate studies. Multiple sequence alignment using Clustal Omega program (https://www.ebi.ac.uk/Tools/msa/clustalo/; accessed on 19 May 2021) was used build a phylogenetic tree representing relative homology between different flavivirus envelope proteins.

Since the purified human C1q cross-reacts with immune-complexes containing non-human primate antibodies, samples from non-human primates infected with each DENV serotype were used to determine dynamics of complement-fixing antibody production following primary infection. The results indicated that the antibody levels trended higher against the infecting serotype, especially at day 341 (Figure 5). Antibodies produced following primary infection also induced complement fixation against heterologous serotypes (Figure 5), and the levels of inter-serotype cross-reactivity could be explained by amino acid homology of the envelope protein among all DENV serotypes (Table 4 and Figure 6). 

### 2.5. Antibodies Produced in Response to Zika Virus Exposure Fix Complement on DENV Structural Proteins

**Figure 7:**
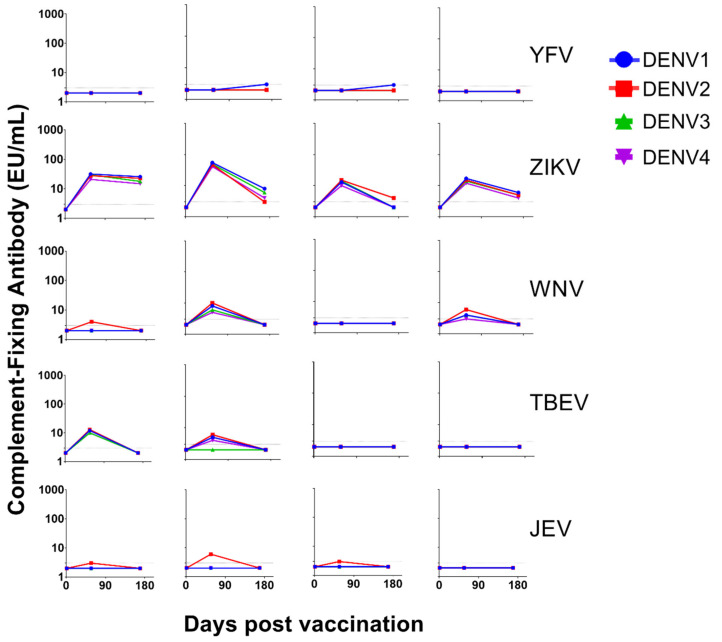
Complement-fixing antibody responses cross-reactive to dengue virus (DENV) in non-human primates immunized with yellow fever (YFV), Zika virus (PIZV), West Nile virus (WNV), tick-borne encephalitis virus (TBEV), and Japanese encephalitis virus (JEV) vaccines. Vaccine used is shown on the right side of the kinetic curves. Complement-fixing antibody kinetic curves (at days 1, 57, and 169) against DENV1, DENV2, DENV3, and DENV4 are shown in blue, red, green, and purple, respectively. Each graphic represents the kinetics of complement-fixing antibody responses against all four DENV serotypes in each animal analyzed in the study. Concentration of 3 EU/mL is shown as dotted line for reference.

Finally, we investigated whether antibodies produced in response to exposure to other *Flaviviruses* can fix complement on DENV structural proteins. Samples collected from Rhesus macaques pre (day 1) and post (days 57 and 169) vaccination with either yellow fever virus (YFV), Zika virus (ZIKV), West Nile virus (WNV), Japanese encephalitis virus (JEV), or tick-borne encephalitis virus (TBEV) vaccines or candidate vaccines were used to determine cross-reactive anti-DENV complement-fixing antibody levels. All animals developed an IgG response upon vaccination (Young et al., manuscript in preparation). Most of the flavivirus vaccines either did not fix (YFV) or inconsistently fixed (WNV, JEV, and TBEV) complement on DENV proteins (Figure 7). Only antibodies produced in response to ZIKV vaccination induced cross-reactive antibodies that fixed complement on all four DENV serotypes in all time points analyzed (Figure 7), which is consistent with envelope protein homology between ZIKV and DENV (Table 4 and Figure 6).

## 3. Discussion

We developed and characterized a novel, reliable, and easy to execute multiplex anti-DENV complement-fixing antibody assay based on the Luminex platform. Using functional purified human C1q and polyclonal antibodies, the assay simultaneously and reproducibly quantifies serum antibodies against structural proteins of all four DENV serotypes that are able to fix complement via the classical pathway. The assay format and optimized parameters were designed to minimize direct interaction between C1q and DENV antigens, which has been observed in other assays and can compromise the complement-fixing antibody assay specificity [9]. 

Antibody-driven CS activation leads to deposition (fixation) of complement proteins, including C3d [18], on the surface of pathogens that facilitates antigen uptake by B cells and follicular dendritic cells (FDC) in germinal centers, mediated by complement receptor CR2 (CD21; [22]). This interaction regulates B cell differentiation by lowering the threshold of activation, promoting proliferation, somatic hypermutation, and class switching as well as helping to maintain effector and memory phenotypes [7]. Thus, C3d deposition can influence antibody production associated with B cell immunity [23]. Complement-fixing antibodies, detected by the interaction between C1q and DENV VLP–antibody immunocomplexes, showed high correlation with C3d deposition, indicating that the assay readout may not only measure the ability of antibodies to fix complement, but also the associated downstream deposition of C3d that can mediate B cell activation. 

Antibodies that can activate CS have been used as a diagnostic biomarker, and more recently implicated in protection against DENV and other *Flaviviruses* by facilitating neutralization, inactivation, and clearance [5,24,25,26]. Previous complement fixation assays have been used to characterize humoral immune responses following vaccination or natural exposure by several *Arbovirus*, including chikungunya virus [27], Japanese encephalitis virus (JEV; [28,29]), Zika virus (ZIKV; [12]), yellow fever virus (YFV; [11,12]), and DENV [14,30,31]. These assays are cumbersome and require highly trained and experienced personnel [15]. Compared with neutralizing antibody assays, traditional complement-fixation assays have low sensitivity and therefore limited value for sero-epidemiological studies [13,15]. Since complement activation contributes to the mechanism of virus neutralization by antibodies [16], low assay sensitivity relative to a neutralization assessment was somewhat unexpected. The anti-DENV complement-fixing antibody assay characterized here demonstrated high sensitivity and specificity (high concordance) in determining DENV serostatus relative to a microneutralization assay (MNT_50_), using a simpler and faster assay format as compared to historical complement fixation assays. The samples used to characterize the anti-dengue virus complement-fixing antibody assay were collected in dengue endemic areas and characterized using a validated MNT_50_ assay that has been used to determine DENV serostatus in the phases II and III clinical trials of Takeda’s attenuated tetravalent dengue vaccine candidate TAK-003 [19,20,32,33]. In a large phase III clinical trial, TAK-003 elicited neutralizing antibody response, as determined by MNT_50_, in 99.5% of seronegative individuals, indicating the assay is suitable for evaluating vaccine-driven seroconversion [34]. The geometric mean and confidence intervals were similar between DENV2 and DENV3 indicating an overall consistent pattern between MNT_50_ and anti-dengue virus complement-fixing assay. However, in few instances we observed that MNT_50_ maximum titers differed across different serotypes (e.g., DENV2 vs. DENV3; Table 2) while complement-fixing antibody levels were relatively constant, which may reflect differences between the populations of antibodies that have complement-fixing and neutralizing antibody functions, as well as the intrinsic variability of the MNT_50_ assay. We also observed DENV serostatus assignment discrepancies between MNT_50_ and complement-fixing antibody assays in a minority of samples. Lack of complement-fixing function in samples MNT_50_ positive (titer ≥10) could be explained by lower total binding IgG levels (<800 RU/mL). Further studies are needed to determine qualitatively and quantitatively the antibody profile associated with complement fixation observed in the assay.

The DENV strains used in MNT_50_ and complement-fixing antibody assays have similar envelope amino acid sequences (percent identity of 97% for DENV1 and DENV3, 100% DENV2 and 99% for DENV4), making antigenic mismatch an improbable explanation for the moderate correlation observed between the assays for DENV1, DENV2, and DENV4. Alternatively, differences in antibody profile could potentially impact both assay outcomes. Neutralizing antibodies detected in the MNT_50_ can be of any subclass/isotype with function associated with specificity of their Fab region to neutralizing epitopes in the envelope protein [35,36]. On the other hand, complement-fixing antibodies are mostly IgG1, IgG3, and IgM with function associated with their Fc region that binds avidly to C1q [16]. Future studies will dissect the antibody responses to understand the role of antibody profiling for the detection of complement-fixing function in the assay. Altogether, as different immunoglobulin isotypes and IgG subclasses may function differently in neutralization and complement fixation, employing both assays may provide a more comprehensive evaluation of the range of functional antibody responses. 

Relatively high homology (66–78%) of the envelope protein leads to cross-reactivity among the four DENV serotypes [37]. Upon primary infection, dominant neutralizing antibody titers are produced in response to the homologous DENV serotype [38] indicating the production of serotype-specific (TS) antibodies. Cross-reactive (CR) antibodies are also produced following first DENV exposure which also contribute to virus neutralization, especially following a second and subsequent infections [39,40,41,42,43]. We identified a positive relationship between neutralizing and complement-fixing antibody levels in seropositive subjects, although it was not clear whether TS and/or CR antibodies were responsible for fixing CS on DENV proteins. Overall, we found higher complement-fixing antibody levels against the infecting serotype consistent with the presence of TS antibodies in non-human primates infected with each virus serotype, which are known to exhibit similar antibody response patterns to humans [44]. However, monotypic infection also generated antibodies that fixed CS on heterologous serotype, which indicates that CR antibodies are also functional in activating CS. Both TS and CR antibodies fixed complement even at 341 days post infection irrespective of virus serotype, which is consistent with preliminary analysis of persistence of this antibody effector function 1 year after TAK-003 vaccination (Nascimento et al., manuscript in preparation). Notably, the complement-fixing response was lower after natural infection with wild type DENV4 than other virus serotypes, possibly due to shorter viremia duration observed in the first 2 weeks post infection (data not shown). The complement-fixing antibody hierarchy was consistent with envelope protein homology, suggesting that, as expected, the major surface protein is the primary target of complement-fixing antibodies. Due to lack of prior DENV infection history from baseline seropositive DEN-203 and DEN-204 study participants as well as the small number of animals studied with known infection history, it was not possible to determine whether the multiplex anti-DENV complement-fixing antibody assay could differentiate the antibody responses to each DENV serotype. Nevertheless, the variability of the envelope protein among DENV serotypes, as well as variability in epitope specificity of immune responses after primary and secondary DENV infections [45] supports use of the multiplex assay format employing antigens from all four DENV serotypes.

The emergence of ZIKV in the Americas in 2015 was followed by a transient period of low DENV incidence from 2017 to 2018 [46,47], supporting the hypothesis that immunity to ZIKV is cross-protective against DENV infection [48,49,50]. This scenario is not unprecedented since ZIKV and DENV prevalence has been suggested to modify clinical YFV infection and the natural cycle between humans and non-human primates with immunologic cross-protection as a possible explanation for the absence of YFV in Asia, coastal Kenya, and the Zika forest [12]. However, the immune interaction among *Flaviviruses* may be more complex than expected. A DENV2 outbreak of unprecedented magnitude was reported in Nicaragua in 2019 and its severity was linked to previous ZIKV exposure [51], yet a similar pattern was not observed in other countries heavily affected by the outbreak [52]. The circumstances by which ZIKV immunity might confer protection against DENV or mediate pathogenesis are not entirely clear, although rapid waning of antibody responses may contribute to these different outcomes [53,54]. In non-human primates immunized with different flavivirus vaccines, only the inactivated ZIKV vaccine candidate (PIZV) elicited a consistent complement-fixing antibody response against DENV, confirming the existence of immunological cross-reactivity between DENV and ZIKV. The clinical impact of this cross-reactivity was not part of the study goals and, therefore, will need further investigation. From a diagnostic perspective, immunological cross-reactivity limits the use of the anti-dengue virus complement-fixing antibody assay to differentiate DENV from ZIKV immunity in areas where both viruses co-circulate. Other than transient cross-reactivity early post-vaccination, antibodies against other flavivirus vaccines did not induce complement-fixation onto DENV structural proteins. This suggests lack of cross-reactivity between DENV and YFV, West Nile virus, tick-borne encephalitis virus, or JEV complement-fixing antibodies. Additional studies are necessary to understand kinetics of potential cross-reactivity of complement-fixing antibodies produced against other *Flaviviruses* following natural infection.

In conclusion, the complement-fixing antibody assay characterized here measures serum antibodies that fix complement on DENV structural proteins, the first step towards CS activation via the classical pathway. The high concordance between neutralizing antibody and complement-fixing antibody assay results indicates either assay can be used to define baseline serostatus after natural DENV exposure. Compared with cell-based neutralization assays, the Luminex based complement-fixing antibody assay has lower variability, is easier and faster to perform, providing results against all DENV serotypes in a single assay within hours instead of days. However, neither the neutralizing [55] nor complement-fixing antibody assays targeting DENV structural protein can differentiate DENV vs. ZIKV infection. 

Complement-fixation has been shown to increase the potency of neutralizing antibodies and to inhibit antibody-dependent enhancement of flavivirus infection [5,24,25,26]. Complement-fixing antibodies have been associated with protective immunity against other pathogens, including *Plasmodium falciparum*, human immunodeficiency virus (HIV), and influenza [56,57,58,59]. Development of a robust complement-fixing antibody assay format will facilitate evaluation of the specificity, magnitude, and function of complement-fixing antibodies in protective immunity against *Flaviviruses* and other pathogens. 

## 4. Materials and Methods

### 4.1. Ethical Statement

Informed assent or consent forms, and the study protocol and its amendments were reviewed and approved by applicable institutional review boards, independent ethics committees, and health authorities. The clinical trials were conducted in accordance with the Declaration of Helsinki [60], and the ICH harmonized tripartite guidelines for Good Clinical Practice [61].

Macaques were screened for antibodies against *Flaviviruses*, and were negative for Herpes B Virus, Simian Retrovirus, Simian Immunodeficiency Virus, Simian T Lymphotropic Virus, *Mycobacterium tuberculosis*, Simian Varicella Virus, Malaria, Salmonella, Shigella, Yersinia, and internal parasites. Macaques were housed at either Wisconsin National Primate Research Center or Inotiv. All in-life practices, including husbandry and environmental enrichment, were approved and conducted per the Institutional Animal Care and Use Committee protocols (protocol number G005401 approved on 27 July 2016 or 2384–14376 approved on 8 June 2018, respectively). Animals were evaluated at least twice daily for clinical signs following vaccinations and challenge. Cage side observations included observation for mortality, moribundity, general health, and signs of toxicity. Treatment with the vaccines had no effect on mortality, physical examinations, cage side observations, body weights, or body weight changes.

### 4.2. Pre-Vaccination Samples from Human Clinical Trials in Dengue Endemic Areas

Pre-vaccination samples collected from participants in two phase 2 double-blind, randomized, placebo controlled clinical trials, DEN-203 (Clinicaltrials.gov: NCT01511250) and DEN-204 (Clinicaltrials.gov: NCT02302066), conducted in dengue endemic areas, were used to investigate the sensitivity and specificity of the anti-dengue virus complement-fixing antibody assay. Trial DEN-203 was conducted in Puerto Rico, Colombia, Singapore, and Thailand to investigate safety and immunogenicity of Takeda’s live-attenuated tetravalent dengue vaccine TAK-003 in pediatric and adult volunteers (aged 1.5–45 years) [19]. Trial DEN-204 was conducted in the Dominican Republic, Panama, and the Philippines, to investigate safety and immunogenicity of TAK-003 in pediatric and adolescent participants (2 to <18 years of age) [20]. Baseline DENV serostatus of individuals was determined using a dengue microneutralization assay. Baseline seronegative was defined as a baseline reciprocal neutralizing titer of <10 for all four dengue serotypes, and baseline seropositive defined as a baseline reciprocal neutralizing titer of ≥10 for at least one dengue serotype.

### 4.3. Commercial Samples from Dengue Immune Subjects

De-identified DENV immune serum samples collected from healthy adults from dengue-endemic areas in Colombia from 2015 to 2016 were purchased from ABO Pharmaceuticals. These samples were used to determine the relationship between complement-fixing antibody response based on C1q fixation and C3d deposition. 

### 4.4. Non-Human Primate Studies

To evaluate anti-DENV complement-fixing antibody production following primary DENV infection with each serotype, adult male *Cynomolgus macaques* 5–7 years, weighing 6–9 kg of Mauritius origin, and naïve to DENV (Bioculture Ltd., Senneville, Maurtitus), were infected subcutaneously with 1 × 10^5^ pfu/0.5 mL of either wild type DENV1 (strain Western Pacific, NIH), DENV2 (strain New Guinea C; NIH), DENV3 (strain Sleman/78; NIH), or DENV4 (strain 1228; CDC) on day 0. Blood samples were collected before and after infection (days 69 and 341) and used for testing of the generation of complement-fixing antibody. All in-life practices followed Institutional Animal Care and Use Committee (IACUC) protocol G005269.

Anti-DENV complement fixing antibodies were also measured in blood samples collected at days 1, 57, and 169 from 2–3 year-old (weighing 3–6 kg) male flavivirus naïve Indian *Rhesus macaques* (*n* = 4 per group) immunized subcutaneously with one dose (at day 1) of 1000 international units of live-attenuated yellow fever vaccine (Stamaril^®^; Sanofi Pasteur, Lyon, France; batches P3B142V and P3D781V) or two doses (at days 1 and 29) of either 10 µg of purified inactivated Zika vaccine candidate (PIZV; batch Z46-006; Takeda), 6 AU (≤460 ng) of inactivated Japanese Encephalitis virus vaccine (Ixiaro^®^, Valneva Scotland Ltd., Livingston, United Kingdom; batches JEV16G32B, JEV17A43B and JEV16H37B), 0.5 mL of inactivated West Nile virus vaccine (Innovator^®^; Fort Dodge, Zoetis, Kalamazoo, MI, USA; batch 194459A), or 1.5 µg of inactivated tick-borne encephalitis virus vaccine (Encepur^®^; GlaxoSmithKline, Bavarian Nordic, Copenhagen, Denmark; batch 190021A). All in-life practices followed IACUC protocol 2384-14376 [62]. 

### 4.5. Coupling of VLPs Expressing Dengue Virus Structural Proteins onto Luminex Magnetic Microspheres

All virus-like particles (VLPs) expressing DENV structural proteins were produced in HEK293 cells and purchased from Native Antigen Company. DENV1 VLP was derived from DENV1 strain Puerto Rico/US/BID-V853/1998 (GenBank accession No. EU482592.1). DENV2 VLP was derived from DENV2 strain Thailand/16681/84 (EMBL-EBI accession No: U87411.1). DENV3 VLP was derived from DENV3 strain Sri Lanka D3/H/IMTSSA-SRI/2000/1266 (GenBank accession No. AY099336.1). DENV4 VLP was derived from DENV4 strain Dominica/814669/1981 (EMBL-EBI accession No: AF326825.1). 

VLPs were coupled to MagPlex^®^ microspheres (Luminex Corporation, Austin, TX, USA) according to the manufacturer recommendations. Briefly, microspheres were washed with distilled water and activated in the presence of 0.1 M sodium phosphate (monobasic) pH 6.2 buffer, 5 mg/mL of N-hydroxysulfosuccinimide (Sulfo-NHS; Thermo Fisher Scientific, Rockford, IL, USA), and 5 mg/mL of 1-Ethyl-3-(3-dimethylaminopropyl) carbodiimide (EDC; Thermo Fisher Scientific, Rockford, IL, USA) for 20 min at room temperature (RT; 18–24 °C). The microspheres were then washed twice with 50 mM 2-(N-(morpholino ethanesulfonic acid (MES) buffer (Boston Bioproducts, Ashland, MA, USA) and incubated for 2 h at RT with each VLP at 5µg per million microspheres ratio. VLP-coupled microspheres were washed and resuspended with 1% (*v*/*v*) bovine serum albumin (BSA) solution prepared in 1X PBS from blocker bovine serum albumin (BSA) 10% (Fisher Scientific, Rockford, IL, USA). VLP-coupled microspheres were counted using a cell counter, Countess II (Invitrogen, Waltham, MA, USA), and stored at 2–8 °C in the dark until use.

### 4.6. Anti-DENV Complement-Fixing Antibody Luminex Assay 

The anti-DENV complement-fixing antibody Luminex assay uses the Luminex multiplex platform to concomitantly quantitate antigen-specific serum antibodies against structural proteins of all four DENV serotypes expressed on VLPs. All incubations were carried out at room temperature (RT; 18–24 °C) on a plate shaker (Heidolph) at 600 rotations per minute (rpm). All reagents and samples were prepared in assay buffer (1% BSA (*v*/*v*) in 1x PBS by diluting the blocker bovine serum albumin (BSA) 10% (Fisher Scientific) 10-fold in 1x PBS solution). The assay was conducted in a 96-well black flat-bottom non-protein binding plate (Corning, Tewksbury, MA, USA). Each plate included a serially diluted reference standard prepared in-house, a negative control serum (Bioreclamation, Westbury, NY, USA ), and three positive (high, mid or low) control samples (ABO Pharmaceuticals, San Diego, CA, USA), in addition to three test samples. Reference, controls, and test samples were heat-inactivated at 56 °C for 30 min prior to serial dilution to inactivate endogenous C1q and other serum factors that might vary in concentration among different sera and thus introduce assay variability. As summarized in Supplemental Figure S10A, 50 µL of each VLP-coupled microspheres at 25 microspheres/µL were combined with 50 µL of 2-fold serially diluted (from 1:2.5 through 1:320) serum samples, incubated for 1 h and washed twice with PBS supplemented with 0.05% (*v*/*v*) tween 20 (PBS-T) using a plate washer (BioTek, model ELx405, Winooski, VT, USA). VLP-coupled microspheres were settled with the aid of a magnetic plate following all washing steps. The antigen–antibody immunocomplexes were detected by adding 50 µL/well of plasma-derived purified human C1q (Quidel, Athens, OH, USA) at 4 µg/mL and incubated for 30 min. Following two washes with PBS-T, the microspheres fixed C1q was detected by adding 50 µL/well of a polyclonal sheep IgG anti-human C1q (Biorad, Kidlington, United Kingdom) at 6.4 µg/mL and incubated for 30 min. After two additional washes with PBS-T, the microspheres were incubated with 50 µL/well of a reporter antibody anti-sheep IgG conjugated to phycoerythrin (Jackson Immunoresearch West Grove, PA, USA) at 10 µg/mL for 30 min. For the final step, the microspheres were then washed twice with PBS-T, reconstituted with 100 µL/well of assay buffer, and read on a Magpix plate reader (Luminex Corporation, Austin, TX, USA). Representative titration curves of the reference and assay controls are shown in Supplemental Figure S10B.

Anti-dengue virus complement-fixing antibody concentrations were calculated relative to a reference standard with an assigned concentration (determined as 50% effective concentration (EC50) against each DENV serotype) for each of the four serotypes in effective units (EU)/mL; 468 (DENV1 VLP), 345 (DENV2 VLP), 369 (DENV3 VLP), and 257 (DENV4 VLP). A 4PL non-linear regression curve of the Luminex fluorescence signal against a log_10_-transformed serum dilution was generated from the reference standard. The fluorescence signal of reference giving 25% of top signal was calculated and used to interpolate the serum dilution from the reference, control, and sample curves. The ratios of these serum dilutions of the samples and controls against the reference were then used to calculate complement-fixing antibody concentration. Final concentrations were generated by multiplying by any serum pre-dilution factor.

### 4.7. Complement C3d Deposition Luminex Assay 

This assay was developed to confirm the results of the anti-DENV complement-fixing antibody assay. Assay incubation conditions (temperature and shaking), buffer, heat inactivation, and microsphere counting were similar to the anti-DENV complement-fixing antibody Luminex assay, unless otherwise noted below. In total, 50 µL of VLP-coupled microspheres were combined with 50 µL of 2-fold serially diluted (from 1:2.5 through 1:320) serum samples, incubated for 1 h and washed twice with PBS supplemented with 0.05% (*v*/*v*) tween 20 (PBS-T) using a plate washer (BioTek, model ELx405, Winooski, VT, USA). The antigen–antibody immunocomplex was detected by adding 50 µL/well of complement-competent human serum (Complement Technology, Tyler, TX, USA) diluted 160-fold in 1x PBS and incubating for 15 min at 37 °C. Following two washes with PBS-T, C3d deposition on VLP-coupled microspheres by antigen-specific serum antibodies was detected by adding 50 µL/well of mouse IgG1 monoclonal antibody anti-human C3d (Quidel, Athens, OH, USA) at 1.25 µg/mL and incubating for 30 min. After two additional washes with PBS-T, microspheres were incubated with 50 µL/well of a reporter antibody anti-mouse IgG conjugated to phycoerythrin (Jackson Immunoresearch, West Grove, PA, USA) at 10 µg/mL for 30 min. At the end, the microspheres were washed twice with PBS-T, reconstituted with 100 µL/well of assay buffer, and read on a Magpix plate reader (Luminex Corporation, Austin, TX, USA). Assay format and titer calculations were also similar to those described for the anti-dengue virus complement-fixing antibody Luminex assay.

### 4.8. Total Binding IgG ELISA

To quantitate total binding IgG specific to DENV-1, DENV-2, DENV-3, and DENV-4, an antigen capture ELISA was used as described elsewhere [63]. Briefly, 96-well MaxiSorp microplates (Nunc, Roskilde, Denmark) were coated with monoclonal antibody clone 4G2 (Absolute Antibody, Boston, MA, USA) during overnight at 4 °C. Microplates were blocked with SuperBlock T20 (Thermo Scientific, Waltham, MA, USA) for 1 h at 37 °C, washed with 1x PBS 0.1% (*v*/*v*) Tween 20 (PBS-T) and incubated with concentrated monovalent tetravalent dengue vaccine (TDV)-1 (strain 16007; GenBank accession No. AAF59976.1), TDV-2 (strain 16681; Uniprot accession No. P29990.1), TDV-3 (strain 16562; Uniprot accession No. A0A173DS74), or TDV-4 (strain 1036; Uniprot accession No. A0A1Z1XCD1) viruses for 1.5 h at 37 °C. The concentration of TDV antigens used in the assay was measured indirectly by serially diluting each TDV virus against a standard concentration of anti-DENV serum in a modified version of the Total Binding IgG Assay. Based on the generated sigmoidal dose–response curve, the dilution point that corresponded to an optical density (OD) 450 value of 2.0 was established as the optimal saturating antigen concentration for coating. Following washes with PBS-T, serially diluted serum samples were then added to the microplate and incubated for 1 h at 37 °C. Following another washing cycle, HRP conjugated anti-IgG secondary antibody (Abcam, Waltham, MA, USA; goat anti-human IgG Fc preadsorbed) was added to the microplate, incubated for 1 h at 37 °C. Microplates were washed and developed with ABTS peroxidase substrate (Seracare, Milford, MA, USA) for 15 min at room temperature. The reaction was stopped with 1x ABTS stop solution (Seracare Milford, MA, USA) and microplates were read at 405 nm using SpectraMax Plus 384 microplate reader (Molecular Devices, San Jose, CA, USA). DENV-specific antibody level was calculated relative to a reference standard using GraphPad Prism version 7 (Graph Pad Software, Inc., San Diego, CA, USA).

### 4.9. Dengue Microneutralization Assay

A dengue microneutralization assay was used to measure neutralizing antibodies against all DENV serotypes (Powel et al., manuscript in preparation, [32]). Heat inactivated serum and virus (the same strains used in the total binding IgG assay) were combined and neutralization occurred overnight at 2–8 °C. Serum/virus mixture was inoculated in triplicates on Vero cells plated on 96-well clear flat bottom tissue-culture-treated microplates (Falcon, Corning, NY, USA), incubated for 90 min at 37 °C, and overlayed with 1% (*w*/*v*) methyl cellulose in DMEM. The virus-exposed cells were then incubated at 34 °C ± 2 °C, 5 ± 3% CO_2_, and fixed. Immunofoci were developed using anti-dengue antibody peroxidase-conjugated secondary antibody (incubated at 37 °C ± 2 °C for 90–120 min) and 2-amino-9-ethyl carbazole (Sigma, St. Louis, MO, USA). The antibody titer of each sample was determined by counting the immunofoci in individual wells.

### 4.10. Anti-DENV Complement-Fixing Antibody Assay Characteristics

The limit of detection (LOD), limit of quantitation (LLOQ), linearity, and precision are described in the Supplemental Material. LOD and LLOQ were determined per Quinn et al. [64].

### 4.11. Homology of Flavivirus Envelope Protein 

Multiple sequence alignment and phylogenetic tree analysis of the envelope amino acid sequence of DENV1 (strain Western Pacific; GenBank accession No. AAN06981.1), DENV2 (strain New Guinea C; GenBank accession No. AAP73862.1), DENV3 (strain Sleman/78; GenBank accession No. AAT69740.1), or DENV4 (strain 1228; GenBank accession No. AER00190.1), Zika virus (strain PRVABC59; GenBank accession No. AMZ03556.1), yellow fever virus (strain Stamaril^®^, Sanofi Pasteur, Lyon, France; GenBank accession No. QGN18661.1), West Nile virus (strain NY99; GenBank accession No. AAG02038.1), Japanese encephalitis virus (strain SA 14-14-2; GenBank accession No. ARE67893.1), and tick-borne encephalitis virus (strain K23; GenBank accession No. AAC62093.1) were carried out using web interface for Clustal Omega and protein blast available at https://www.ebi.ac.uk/Tools/msa/clustalo/ (accessed on 19 May 2021) and https://blast.ncbi.nlm.nih.gov/Blast.cgi# (accessed on 19 May 2021), respectively. The former was used to construct a phylogenetic tree, while the latter, to calculate protein percent identity.

### 4.12. Statistical Analysis

Specificity of anti-DENV complement-fixing antibodies were calculated based on the percentage of samples with MNT_50_ < 10 (seronegative) that were below the threshold titer of 3 EU/mL. On the other hand, sensitivity was calculated as the percentage of samples with MNT_50_ ≥10 (seropositive) that were equal to or above the threshold titer of 3 EU/mL. 

The receiver operating characteristic (ROC) curves were created by plotting the true seropositive rate (defined as the rate of complement-fixing antibodies with titers equal to or above the threshold titer of 3 EU/mL in seropositive samples (MNT_50_ ≥ 10)) against the false seropositive rate (defined as complement-fixing antibody titers equal or above the threshold titer of 3 EU/mL in samples considered seronegative (MNT_50_ < 10)) at various complement-fixing antibody thresholds from <3 EU/mL to approximately 1000 EU/mL. The area under the ROC was calculated as a single numerical measurement to describe the performance of the complement-fixing antibody titers as a biomarker to classify dengue serostatus.

Correlation analysis between complement-fixing antibody titers and microneutralization titers or total binding IgG concentrations was carried out using log_10_-transformed values using JMP version 13.1.0 software (SAS Institute Inc., San Diego, CA, USA). Samples were color-coded based on DENV serostatus (blue—seronegative; red—seropositive). Correlation analysis between complement-fixing and C3d deposition antibody concentrations was carried out using log_10_-transformed values using GraphPad Prism version 8.1.0 (GraphPad Software, Inc., San Diego, CA, USA).

## Figures and Tables

**Table 1:** Demographics of participants enrolled in DEN-203 and DEN-204 clinical trials analyzed for presence of neutralizing and complement-fixing antibodies at day 1 only (pre-vaccination).

Study	*n*	Number of Samples per Age Groups (Years)			
		1–5	6–11	12–20	>20
DEN-203	37	8	12	10	7
DEN-204	16	6	8	2	0
Total	53	14	20	12	7

**Table 2:** Neutralizing (MNT_50_) and complement-fixing antibody levels against all four dengue virus serotypes from subjects living in endemic areas. Minimum, maximum, geometric mean (GeoMean), and 95% confidence interval (CI) titers were determined for both MNT_50_ and complement-fixing antibodies. For samples with undetectable complement-fixing antibody levels, minimum concentrations were arbitrarily set to 2 EU/mL for all DENV serotypes. For samples with undetectable MNT_50_ levels, minimum titers were arbitrarily set to 5 for all DENV serotypes.

Virus	MNT50 Titers			Complement-Fixing Antibody Titers (EU/mL)		
	Minimum	Maximum	GeoMean (95% CI)	Minimum	Maximum	GeoMean (95% CI)
DENV1	5	20480	19 (10, 35)	2	1915	5 (3, 7)
DENV2	5	10919	20 (10, 40)	2	1565	4 (3, 7)
DENV3	5	5120	15 (9, 26)	2	1564	4 (3, 7)
DENV4	5	1442	12 (7, 20)	2	730	4 (3, 6)

**Table 3:** Sensitivity and specificity of the complement-fixing antibody assay compared with the microneutralization assay for each dengue virus (DENV) serotype.

Viruses	Number of Samples					
	MNT_50_ ≥ 10	Complement ≥ 3 EU/mL	Sensitivity (%)	MNT_50_ < 10	Complement < 3 EU/mL	Specificity (%)
DENV1	17	14	82%	36	35	97%
DENV2	14	14	100%	39	39	100%
DENV3	16	13	81%	37	37	100%
DENV4	12	11	92%	41	39	95%

**Table 4:** Homology of the envelope amino acid sequence of different *Flaviviruses* used in non-human primate studies. Multiple envelope amino acid sequences were aligned using protein blast (https://blast.ncbi.nlm.nih.gov/Blast.cgi; accessed on 19 May 2021) that was used to determine pairwise protein percent identity. Protein percent identity was color coded where red and green represent high and low similarity between proteins, respectively.

	DENV1	DENV2	DENV3	DENV4	ZIKV	JEV	WNV	YFV	TBEV
**DENV1**	100	69	78	64	59	51	51	43	39
**DENV2**	-	100	69	65	55	48	48	44	38
**DENV3**	-	-	100	63	59	49	48	42	38
**DENV4**	-	-	-	100	57	48	50	40	40
**ZIKV**	-	-	-	-	100	54	54	42	40
**JEV**	-	-	-	-	-	100	77	45	40
**WNV**	-	-	-	-	-	-	100	45	42
**YFV**	-	-	-	-	-	-	-	100	41
**TBEV**	-	-	-	-	-	-	-	-	100

## Data Availability

Data supporting results on the phase II clinical trial samples DEN-203 and DEN-204 can be found on “Nascimento et al. All Data_CFA_MNT50_Total Binding_Supplemental Material.xlsx” file in the Supplemental Materials.

## Supplementary Materials

The following are available online at https://www.mdpi.com/article/10.3390/ijms222112004/s1.

## References

[1] Bachal R., Alagarasu K., Singh A., Salunke A., Shah P., Cecilia D. (2015). Higher levels of dengue-virus-specific IgG and IgA during pre-defervescence associated with primary dengue hemorrhagic fever. Arch. Virol..

[2] Dowd K.A., Pierson T.C. (2011). Antibody-mediated neutralization of flaviviruses: A reductionist view. Virology.

[3] Vázquez S., Cabezas S., Pérez A., Pupo M., Ruiz D., Calzada N., Bernardo L., Castro O., González D., Serrano T. (2007). Kinetics of antibodies in sera, saliva, and urine samples from adult patients with primary or secondary dengue 3 virus infections. Int. J. Infect. Dis..

[4] Della-Porta A.J., Westaway E.G., Robbins S.J., Bussell R.H., Rapp F. (1978). A Multi-Hit Model for the Neutralization of Animal Viruses. J. Gen. Virol..

[5] Mehlhop E., Nelson S., Jost C.A., Gorlatov S., Johnson S., Fremont D.H., Diamond M.S., Pierson T.C. (2009). Complement Protein C1q Reduces the Stoichiometric Threshold for Antibody-Mediated Neutralization of West Nile Virus. Cell Host Microbe.

[6] Yamanaka A., Kosugi S., Konishi E. (2008). Infection-Enhancing and -Neutralizing Activities of Mouse Monoclonal Antibodies against Dengue Type 2 and 4 Viruses Are Controlled by Complement Levels. J. Virol..

[7] Carroll M.C. (2008). Complement and humoral immunity. Vaccine.

[8] Cooper N.R. (1985). The Classical Complement Pathway: Activation and Regulation of the First Complement Component. Adv. Immunol..

[9] Douradinha B., McBurney S.P., de Melo K.M.S., Smith A.P., Krishna N.K., Barratt-Boyes S.M., Evans J.D., Nascimento E.J., Marques E.T. (2014). C1q binding to dengue virus decreases levels of infection and inflammatory molecules transcription in THP-1 cells. Virus Res..

[10] Farrell K.T. (1978). An epidemic of dengue fever in Wewak. Papua New Guin. Med. J..

[11] Monath T.P., Craven R.B., Muth D.J., Trautt C.J., Calisher C.H., Fitzgerald S.A. (1980). Limitations of the Complement-Fixation Test for Distinguishing Naturally Acquired from Vaccine-Induced Yellow Fever Infection in Flavivirus-Hyperendemic Areas. Am. J. Trop. Med. Hyg..

[12] Monath T.P., Wilson D.C., Casals J. (1973). The 1970 yellow fever epidemic in Okwoga District, Benue Plateau State, Nigeria. 3. Serological responses in persons with and without pre-existing heterologous group B immunity. Bull. World Health Organ..

[13] Rice C.E. (1960). The Use of Complement-Fixation Tests in the Study and Diagnosis of Viral Diseases in Man and Animals—A Review, V. The Arborviruses. Can. J. Comp. Med. Vet. Sci..

[14] Sabin A.B., Young I. (1948). A Complement Fixation Test for Dengue. Exp. Biol. Med..

[15] De Paula S.O., Fonseca B. (2004). Dengue: A review of the laboratory tests a clinician must know to achieve a correct diagnosis. Braz. J. Infect. Dis..

[16] Bernet J., Mullick J., Singh A.K., Sahu A. (2003). Viral mimicry of the complement system. J. Biosci..

[17] Sörman A., Zhang L., Ding Z., Heyman B. (2014). How antibodies use complement to regulate antibody responses. Mol. Immunol..

[18] Toapanta F.R., Ross T.M. (2006). Complement-Mediated Activation of the Adaptive Immune Responses: Role of C3d in Linking the Innate and Adaptive Immunity. Immunol. Res..

[19] Sirivichayakul C., A Barranco-Santana E., Rivera I.E., Kilbury J., Raanan M., Borkowski A., Papadimitriou A., Wallace D. (2020). Long-term Safety and Immunogenicity of a Tetravalent Dengue Vaccine Candidate in Children and Adults: A Randomized, Placebo-Controlled, Phase 2 Study. J. Infect. Dis..

[20] Tricou V., Sáez-Llorens X., Yu D., Rivera L., Jimeno J., Villarreal A.C., Dato E., de Suman O.S., Montenegro N., DeAntonio R. (2020). Safety and immunogenicity of a tetravalent dengue vaccine in children aged 2–17 years: A randomised, placebo-controlled, phase 2 trial. Lancet.

[21] Berrar D., Flach P. (2012). Caveats and pitfalls of ROC analysis in clinical microarray research (and how to avoid them). Briefings Bioinform..

[22] Erdei A., Isaák A., Török K., Sándor N., Kremlitzka M., Prechl J., Bajtay Z. (2009). Expression and role of CR1 and CR2 on B and T lymphocytes under physiological and autoimmune conditions. Mol. Immunol..

[23] Dunn M.D., Rossi S.L., Carter D.M., Vogt M.R., Mehlhop E., Diamond M.S., Ross T.M. (2010). Enhancement of anti-DIII antibodies by the C3d derivative P28 results in lower viral titers and augments protection in mice. Virol. J..

[24] Mehlhop E., Ansarah-Sobrinho C., Johnson S., Engle M., Fremont D.H., Pierson T.C., Diamond M.S. (2007). Complement Protein C1q Inhibits Antibody-Dependent Enhancement of Flavivirus Infection in an IgG Subclass-Specific Manner. Cell Host Microbe.

[25] Mehlhop E., Fuchs A., Engle M., Diamond M.S. (2009). Complement modulates pathogenesis and antibody-dependent neutralization of West Nile virus infection through a C5-independent mechanism. Virology.

[26] Mehlhop E., Whitby K., Oliphant T., Marri A., Engle M., Diamond M.S. (2005). Complement Activation Is Required for Induction of a Protective Antibody Response against West Nile Virus Infection. J. Virol..

[27] Eckels K.H., Harrison V.R., Hetrick F.M. (1970). Chikungunya virus vaccine prepared by Tween-ether extraction. Appl. Microbiol..

[28] Hodes H.L., Thomas L., Peck J.L. (1945). Cause of an Outbreak of Encephalitis Established by Means of Complement-Fixation Tests. Exp. Biol. Med..

[29] Southam C.M. (1956). Serologic Studies of Encephalitis in Japan I. Hemagglutination-inhibiting, Complement-fixing, and Neutralizing Antibody Following Overt Japanese B Encephalitis. J. Infect. Dis..

[30] Rowan L.C. (1959). An outbreak of dengue-like fever, North Queensland, 1954; serological findings with the virus neutralization and complement fixation tests. Med J. Aust..

[31] Smith C.E.G. (1957). A localized outbreak of dengue fever in Kuala Lumpur: Serological aspects. J. Hyg..

[32] Osorio J.E., Velez I.D., Thomson C., Lopez L., Jimenez A., Haller A.A., Silengo S., Scott J., Boroughs K.L., Stovall J.L. (2014). Safety and immunogenicity of a recombinant live attenuated tetravalent dengue vaccine (DENVax) in flavivirus-naive healthy adults in Colombia: A randomised, placebo-controlled, phase 1 study. Lancet Infect. Dis..

[33] Rivera L., Biswal S., Sáez-Llorens X., Reynales H., López-Medina E., Borja-Tabora C., Bravo L., Sirivichayakul C., Kosalaraksa P., Vargas L.M. (2021). Three years efficacy and safety of Takeda’s dengue vaccine candidate (TAK-003). Clin. Infect. Dis..

[34] Biswal S., Reynales H., Saez-Llorens X., Lopez P., Borja-Tabora C., Kosalaraksa P., Sirivichayakul C., Watanaveeradej V., Rivera L., Espinoza F. (2019). Efficacy of a Tetravalent Dengue Vaccine in Healthy Children and Adolescents. N. Engl. J. Med..

[35] Boudreau C., Alter G. (2019). Extra-Neutralizing FcR-Mediated Antibody Functions for a Universal Influenza Vaccine. Front. Immunol..

[36] Wahala W.M.P.B., De Silva A.M. (2011). The Human Antibody Response to Dengue Virus Infection. Viruses.

[37] Venkatachalam R., Subramaniyan V. (2014). Homology and conservation of amino acids in E-protein sequences of dengue serotypes. Asian Pac. J. Trop. Dis..

[38] Guzman M.G., Alvarez M., Rodriguez-Roche R., Bernardo L., Montes T., Vazquez S., Morier L., Alvarez A., A Gould E., Kourí G. (2007). Neutralizing Antibodies after Infection with Dengue 1 Virus. Emerg. Infect. Dis..

[39] Buddhari D., Aldstadt J., Endy T.P., Srikiatkhachorn A., Thaisomboonsuk B., Klungthong C., Nisalak A., Khuntirat B., Jarman R.G., Fernandez S. (2014). Dengue Virus Neutralizing Antibody Levels Associated with Protection from Infection in Thai Cluster Studies. PLoS Negl. Trop. Dis..

[40] Corbett K.S., Katzelnick L., Tissera H., Amerasinghe A., De Silva A.D., De Silva A.M. (2015). Preexisting Neutralizing Antibody Responses Distinguish Clinically Inapparent and Apparent Dengue Virus Infections in a Sri Lankan Pediatric Cohort. J. Infect. Dis..

[41] Gibbons R.V., Kalanarooj S., Jarman R.G., Nisalak A., Vaughn D.W., Endy T.P., Mammen M.P., Srikiatkhachorn A. (2007). Analysis of Repeat Hospital Admissions for Dengue to Estimate the Frequency of Third or Fourth Dengue Infections Resulting in Admissions and Dengue Hemorrhagic Fever, and Serotype Sequences. Am. J. Trop. Med. Hyg..

[42] Katzelnick L.C., Montoya M., Gresh L., Balmaseda A., Harris E. (2016). Neutralizing antibody titers against dengue virus correlate with protection from symptomatic infection in a longitudinal cohort. Proc. Natl. Acad. Sci. USA.

[43] Olkowski S., Forshey B.M., Morrison A.C., Rocha C., Vilcarromero S., Halsey E.S., Kochel T.J., Scott T.W., Stoddard S.T. (2013). Reduced Risk of Disease During Postsecondary Dengue Virus Infections. J. Infect. Dis..

[44] Mohammed Y.S., Grešiková M., Adamyová K., Ragib A.H., El-Dawala K. (1970). Studies on Arboviruses in Egypt: II. Contribution of Arboviruses to the aetiology of undiagnosed fever among children. J. Hyg..

[45] Crill W.D., Hughes H.R., DeLorey M.J., Chang G.-J.J. (2009). Humoral Immune Responses of Dengue Fever Patients Using Epitope-Specific Serotype-2 Virus-Like Particle Antigens. PLoS ONE.

[46] Perez F., Llau A., Gutierrez G., Bezerra H., Coelho G., Ault S., Barbiratto S.B., De Resende M.C., Cerezo L., Kleber G.L. (2019). The decline of dengue in the Americas in 2017: Discussion of multiple hypotheses. Trop. Med. Int. Health.

[47] Ribeiro G.S., Kikuti M., Tauro L.B., Nascimento L.C.J., Cardoso C., Campos G.S., Ko A., Weaver S.C., Reis M.G., Kitron U. (2018). Does immunity after Zika virus infection cross-protect against dengue?. Lancet Glob. Health.

[48] Borchering R.K., Huang A.T., Mier-Y-Teran-Romero L., Rojas D.P., Rodriguez-Barraquer I., Katzelnick L.C., Martinez S.D., King G.D., Cinkovich S.C., Lessler J. (2019). Impacts of Zika emergence in Latin America on endemic dengue transmission. Nat. Commun..

[49] Breitbach M.E., Newman C.M., Dudley D.M., Stewart L.M., Aliota M.T., Koenig M.R., Shepherd P.M., Yamamoto K., Crooks C.M., Young G. (2019). Primary infection with dengue or Zika virus does not affect the severity of heterologous secondary infection in macaques. PLoS Pathog..

[50] Pérez-Guzmán E.X., Pantoja P., Serrano-Collazo C., Hassert M.A., Ortiz-Rosa A., Rodríguez I.V., Giavedoni L., Hodara V., Parodi L., Cruz L. (2019). Time elapsed between Zika and dengue virus infections affects antibody and T cell responses. Nat. Commun..

[51] Katzelnick L.C., Narvaez C., Arguello S., Mercado B.L., Collado D., Ampie O., Elizondo D., Miranda T., Carillo F.B., Mercado J.C. (2020). Zika virus infection enhances future risk of severe dengue disease. Science.

[52] Brito A.F., Machado L.C., Oidtman R.J., Siconelli M.J.L., Tran Q.M., Fauver J.R., Carvalho R.D.D.O., Dezordi F.Z., Pereira M.R., de Castro-Jorge L.A. (2021). Lying in wait: The resurgence of dengue virus after the Zika epidemic in Brazil. Nat. Commun..

[53] Henderson A.D., Aubry M., Kama M., Vanhomwegen J., Teissier A., Mariteragi-Helle T., Paoaafaite T., Teissier Y., Manuguerra J.-C., Edmunds J. (2020). Zika seroprevalence declines and neutralizing antibodies wane in adults following outbreaks in French Polynesia and Fiji. eLife.

[54] Moreira-Soto A., Sampaio G.D.S., Pedroso C., Postigo-Hidalgo I., Berneck B.S., Ulbert S., Brites C., Netto E.M., Drexler J.F. (2020). Rapid decline of Zika virus NS1 antigen-specific antibody responses, northeastern Brazil. Virus Genes.

[55] Montoya M., Collins M., Dejnirattisai W., Katzelnick L.C., Puerta-Guardo H., Jadi R., Schildhauer S., Supasa P., Vasanawathana S., Malasit P. (2018). Longitudinal Analysis of Antibody Cross-neutralization Following Zika Virus and Dengue Virus Infection in Asia and the Americas. J. Infect. Dis..

[56] Co M.D.T., Terajima M., Thomas S.J., Jarman R.G., Rungrojcharoenkit K., Fernandez S., Yoon I.-K., Buddhari D., Cruz J., Ennis F.A. (2014). Relationship of Preexisting Influenza Hemagglutination Inhibition, Complement-Dependent Lytic, and Antibody-Dependent Cellular Cytotoxicity Antibodies to the Development of Clinical Illness in a Prospective Study of A(H1N1)pdm09 Influenza in Children. Viral Immunol..

[57] Lofano G., Gorman M.J., Yousif A.S., Yu W.-H., Fox J.M., Dugast A.-S., Ackerman M.E., Suscovich T.J., Weiner J., Barouch D. (2018). Antigen-specific antibody Fc glycosylation enhances humoral immunity via the recruitment of complement. Sci. Immunol..

[58] Rattan A., Pawar S.D., Nawadkar R., Kulkarni N., Lal G., Mullick J., Sahu A. (2017). Synergy between the classical and alternative pathways of complement is essential for conferring effective protection against the pandemic influenza A(H1N1) 2009 virus infection. PLoS Pathog..

[59] Reiling L., Boyle M., White M.T., Wilson D., Feng G., Weaver R., Opi D.H., Persson K.E.M., Richards J.S., Siba P.M. (2019). Targets of complement-fixing antibodies in protective immunity against malaria in children. Nat. Commun..

[60] Association W.M. Declaration of Helsinki. Ethical Principles for Medical Research Involving Human Subjects. https://wwwwmanet/policies-post/wma-declaration-of-helsinki-ethical-principles-for-medical-research-involving-human-subjects/.

[61] (ICH) ICfHoTRfPfHU. Guideline for Good Clinical Practice E6(R2). https://wwwemaeuropaeu/en/ich-e6-r2-good-clinical-practice.

[62] Young G., Bohning K.J., Zahralban-Steele M., Hather G., Tadepalli S., Mickey K., Godin C.S., Sanisetty S., Sonnberg S., Patel H.K. (2020). Complete Protection in Macaques Conferred by Purified Inactivated Zika Vaccine: Defining a Correlate of Protection. Sci. Rep..

[63] Michlmayr D., Andrade P., Nascimento E.J.M., Parker A., Narvekar P., Dean H.J., Harris E. (2021). Characterization of the Type-Specific and Cross-Reactive B-Cell Responses Elicited by a Live-Attenuated Tetravalent Dengue Vaccine. J. Infect. Dis..

[64] Quinn C.P., Semenova V.A., Elie C.M., Romero-Steiner S., Greene C., Li H., Stamey K., Steward-Clark E., Schmidt D.S., Mothershed E. (2002). Specific, Sensitive, and Quantitative Enzyme-Linked Immunosorbent Assay for Human Immunoglobulin G Antibodies to Anthrax Toxin Protective Antigen. Emerg. Infect. Dis..

