# Supplementary material for: Development and Characterization of a Multiplex Assay to Quantify Complement-Fixing Antibodies against Dengue Virus
Source: Int J Mol Sci. 2021 Nov 5;22(21):12004. doi: 10.3390/ijms222112004 (PMC8584793; doi:10.3390/ijms222112004)
Supplement: Supplementary file 1 [file ijms-22-12004-s001.zip › ijms-1409319-supplementary_Final2 (1).pdf]

Supplemental Material

Complement-fixing antibody assay characterization

In order to estimate limit of detection (LOD) of the assay, reference curves of 4 plates were evaluated. The mean fluorescence intensity (MFI) at each analyte concentration was used to fit the overall curve using the 4-PL non-linear regression model. LOD was calculated by determining the minimum detectable concentration (MDC) and reliable detection limit (RDL) for each DENV serotype according to Quinn et al [64]. MDC is the analyte concentration corresponding to the interpolated intersection of the lower asymptote of the upper 95% confidence band with the 4-PL fit of the average reference curve. The reliable detection limit (RDL) is the lowest concentration of analyte that has high probability of producing a response significantly greater than the response at zero concentration of analyte. The RDL is the concentration of antibody corresponding to the interpolated intersection of the lower asymptote of the upper 95% confidence band with the lower 95% confidence band of the average reference curve. The MDC estimated was used as the LOD of the assay and were 0.25 EU/mL, 0.20 EU/mL, 0.22 EU/mL and 0.14 EU/mL for DENV1, DENV2, DENV3 and DENV4, respectively (Supplemental Figure S2B).

The lower limit of quantitation (LLOQ) was then determined by the minimum concentration at which samples yielded determinations with suitable precision (<20% CV) and accuracy (80% < % Recovery < 120%) [61]. Reference standard at various dilutions was prepared in IgG-depleted human serum (Molecular Innovations) and evaluated in the anti-DENV complement-fixing assay as independent samples (representative sample curves and antibody concentrations are shown on Supplemental Figure 1 and Supplemental Table S1). Complement-fixing antibody concentration was determined for each dilution in 5 independent runs carried out in different days. The expected antibody concentration of each sample was calculated by dividing the complement-fixing antibody level of the undiluted reference standard by the dilution factor used during sample preparation. The complement-fixing antibody concentration for each dilution of the reference (observed concentration) was calculated by interpolating the fluorescence signal equivalent to 25% of the effective dose of the reference standard on each sample titration curve. The interpolated concentration was normalized by the titer of the reference. Percent (%) recovery was calculated with the equation % Recovery = [(average observed concentration)/expected concentration]*100 for each sample. Supplemental Tables S2–S5 show that all samples with detectable complement-fixing antibody levels (dilutions 1:2 through 1:128) had acceptable precision and accuracy. Supplemental Table S6 summarizes the assay LLOQ per DENV serotype.

Assay linearity was evaluated by plotting the mean value of the observed concentration of five independent runs of a positive sample at multiple concentrations on

the anti-DENV complement-fixing antibody assay. Linear regression of the observed and expected concentrations was performed, and the slope with a 95% confidence interval was calculated. Supplemental Figure S3 shows that slope and 95% confidence interval were within the acceptable ranges of between 0.8–1.2.

To evaluate the assay precision, high, medium and low positive control samples used in the complement-fixing antibody assay were tested five times each per run by two operators on two different occasions. For determination of intra-assay precision, the percent coefficient of variation [%CV = Standard deviation/Mean*100] of the complement-fixing antibody concentration was calculated for each sample within each run by each operator. For determination of inter-assay precision, the %CV of the complement-fixing antibody concentration was calculated in between the runs by both operators. Intra- and inter-assay precision, irrespective of DENV serotype was below 21% (Supplemental Figures S4–S6).

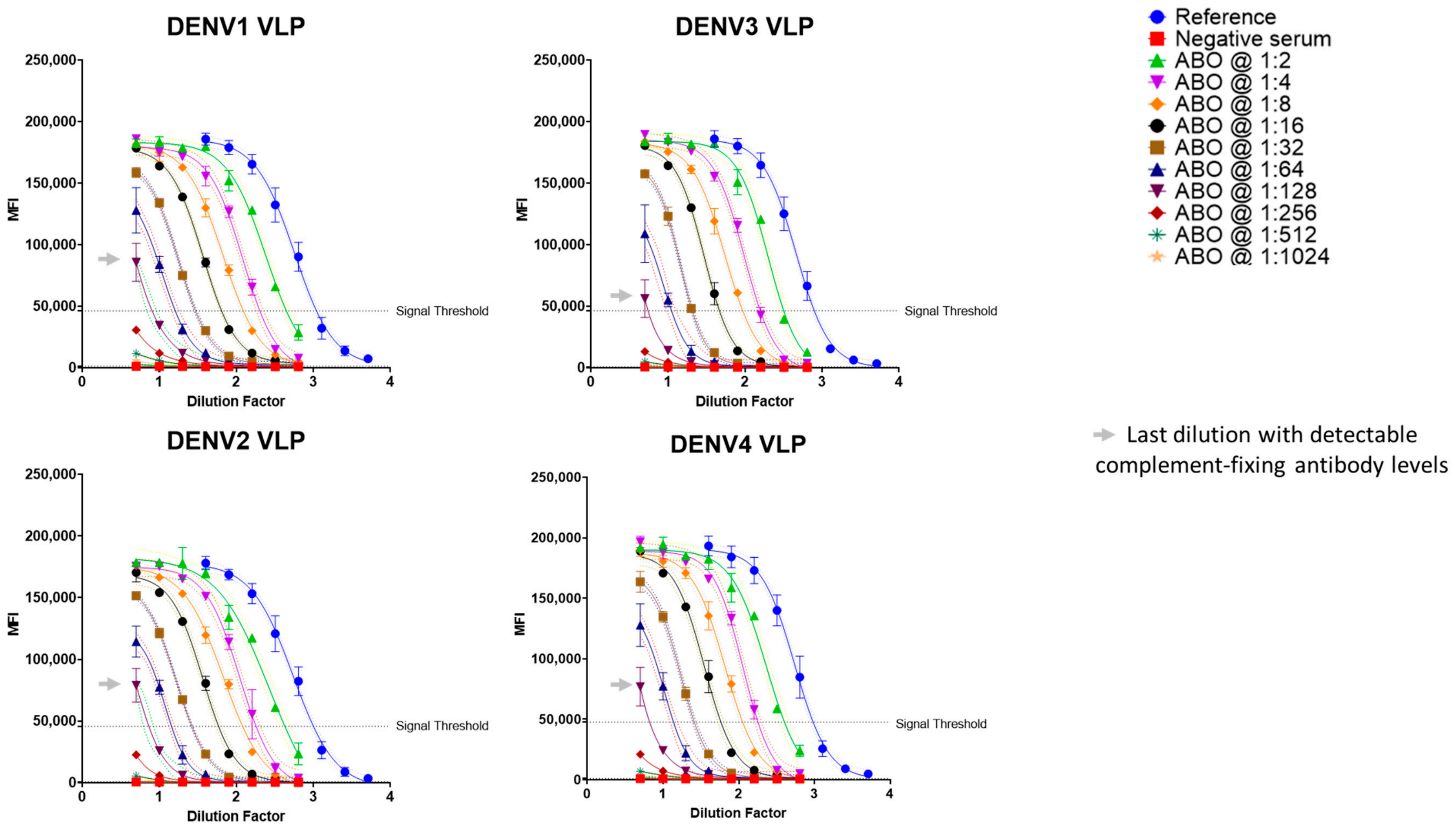

Figure S1. Representative titration curves of reference standard (ABO) at various dilutions prepared in IgG-depleted human serum. Dotted line represents signal threshold equivalent of EC25 of the reference standard determined in each plate/antigen that was interpolated in each sample titration curve to calculate complement-fixing antibody levels. The gray arrow represents the last dilution of the reference standard with detectable complement-fixing antibody levels.

Table S1. Representative titration curves of reference standard (ABO) at various dilutions (1:2 to 1:1024) prepared in IgG-depleted human serum. Gray area in the table represents samples that did not cross the signal threshold and, therefore, have undetectable complement-fixing antibody levels. Expected complement-fixing antibody levels was calculated relative to reference standard assigned concentration at neat. The observed compleement-fixing antibody levels was determined by running each dilution of the reference standard as a sample in the assay.

Samples	Expected Levels Complement-Fixing Antibody (EU/mL)				Observed levels Complement-Fixing Antibodies (EU/mL)			
	**DENV1**	**DENV2**	**DENV3**	**DENV4**	**DENV1**	**DENV2**	**DENV3**	**DENV4**
ABO @ Neat	468.00	345.00	369.00	257.00	-	-	-	-
ABO @ 1:2	234.00	172.50	184.50	128.50	200.55	149.99	155.68	119.37
ABO @ 1:4	117.00	86.25	92.25	64.25	90.68	63.41	77.16	53.10
ABO @ 1:8	58.50	43.13	46.13	32.13	55.46	42.39	45.76	33.47
ABO @ 1:16	29.25	21.56	23.06	16.06	29.83	22.52	22.50	15.88
ABO @ 1:32	14.63	10.78	11.53	8.03	13.69	10.13	10.00	7.07
ABO @ 1:64	7.31	5.39	5.77	4.02	7.48	5.52	5.37	3.75
ABO @ 1:128	3.66	2.70	2.88	2.01	3.53	2.47	2.62	1.89
ABO @ 1:256	1.83	1.35	1.44	1.00	NC	NC	NC	NC
ABO @ 1:512	0.91	0.67	0.72	0.50	NC	NC	NC	NC
ABO @ 1:1024	0.46	0.34	0.36	0.25	NC	NC	NC	NC

Signal threshold was not reached

NC—not calculated

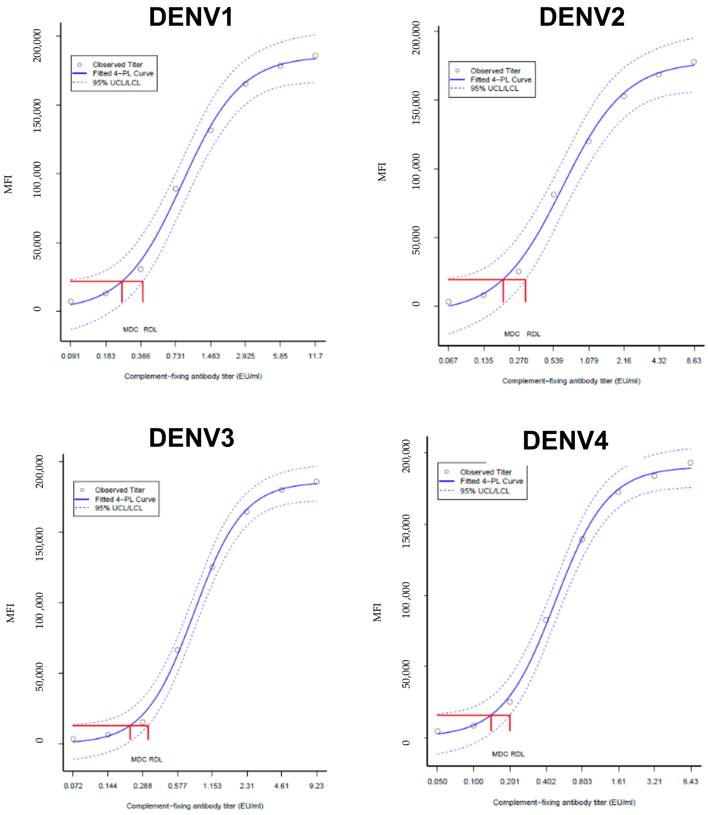

B

Virus	Comeplement-fixing antibody (EU/mL)	
	MDC	RDL
DENV1	0.25	0.38
DENV2	0.20	0.31
DENV3	0.22	0.32
DENV4	0.14	0.20

Figure S2. Limit of detection (LOD) analysis for the anti-dengue virus complement-fixing antibody assay. (**A**) 4PL Curves of the reference standard, minimum detectable concentration (MDC) and reliable detection limit (RDL) determined for each DENV serotype. MDC is the concentration of antibody corresponding to the interpolated intersection of the lower asymptote of the upper 95% confidence band (CB) with the 4-PL fit of the average reference curve. RDL is the concentration of antibody corresponding to the interpolated intersection of the lower asymptote of the upper 95% CB with the lower 95% CB of the average reference curve. (**B**) MDC and RDL values calculated for all DENV serotypes.

Table S2. Characterization of limit of quantitation (LLOQ) of the anti-DENV1 complement-fixing antibody assay. Anti-DENV complement-fixing antibody assay was conducted against all DENV serotypes. LLOQ analysis shown only include DENV1. Complement-fixing antibody concentrations against DENV1 were determined on five different occasions in one sample at multiple dilutions. Expected and averaged observed concentrations, as well as percent (%) recovery and coefficient of variance (%CV; precision) were calculated. Rows highlighted in grey represent samples in which concentrations were calculated based on extrapolation of the titration curves (threshold signal was not reached) and, therefore, were considered not detectable.

Samples	Expected Concentration	Complement-Fixing Antibody Titer (EU/mL) DENV1 VLP (Obtained Concentration)								
		**VBU-01140-184**	**VBU-01140-185**	**VBU-01140-188**	**VBU-01140-190**	**VBU-01140-193**	**Average**	**SD**	**% Recovery**	**%CV**
Reference	468.00	-	-	-	-	-	-	-	-	-
ABO Pool 1:2	234.00	257.16	229.55	184.00	187.70	214.78	214.64	30.39	92%	14%
ABO Pool 1:4	117.00	126.08	121.01	112.95	94.45	94.72	109.84	14.70	94%	13%
ABO Pool 1:8	58.50	64.37	59.34	50.52	49.12	50.84	54.84	6.68	94%	12%
ABO Pool 1:16	29.25	28.31	28.39	23.02	25.29	26.03	26.21	2.25	90%	9%
ABO Pool 1:32	14.63	12.95	14.87	12.77	12.46	12.68	13.15	0.98	90%	7%
ABO Pool 1:64	7.31	7.63	6.95	8.66	5.99	7.78	7.40	1.00	101%	13%
ABO Pool 1:128	3.66	3.69	3.36	4.02	2.90	3.68	3.53	0.43	97%	12%
ABO Pool 1:256	1.83	1.69	1.68	1.79	1.09	1.61	1.57	0.28	NC	18%
ABO Pool 1:512	0.91	0.87	-	-	-	-	0.87	NC	NC	NC
ABO Pool 1:1024	0.46	-	-	-	-	-	NC	NC	NC	NC

The signal threshold was not reached   NC—Not calculated

Table S3. Characterization of limit of quantitation (LLOQ) of the anti-DENV2 complement-fixing antibody assay. Anti-DENV complement-fixing antibody assay was conducted againt all DENV serotypes. LLOQ analysis shown only include DENV2. Complement-fixing antibody concentrations against DENV2 were determined on five different occasions in one sample using multiple dilutions. Expected and averaged observed concentrations as well as percent (%) recovery and coefficient of variance (%CV; precision) were calculated. Rows highlighted in grey represent samples in which concentrations were calculated based on extrapolation of the titration curves (threshold signal was not reached) and, therefore, were considered not detectable.

**Samples**	**Expected Concentration**	**Complement-Fixing Antibody Titer (EU/mL) DENV2 VLP (Obtained Concentration)**								
		**VBU-01140-184**	**VBU-01140-185**	**VBU-01140-188**	**VBU-01140-190**	**VBU-01140-193**	**Average**	**SD**	**% Recovery**	**%CV**
Reference	345.00	-	-	-	-	-	-	-	-	-
ABO Pool 1:2	172.50	216.39	177.48	133.24	140.42	161.12	165.73	33.25	96%	20%
ABO Pool 1:4	86.25	99.61	82.69	81.97	67.33	64.81	79.28	14.00	92%	18%
ABO Pool 1:8	43.13	49.65	39.87	35.38	34.54	34.70	38.83	6.43	90%	17%
ABO Pool 1:16	21.56	22.04	19.70	17.02	18.20	19.40	19.27	1.88	89%	10%
ABO Pool 1:32	10.78	9.22	10.95	9.82	9.23	9.64	9.77	0.71	91%	7%
ABO Pool 1:64	5.39	5.35	5.21	6.48	4.62	6.53	5.64	0.84	105%	15%
ABO Pool 1:128	2.70	2.78	2.55	2.97	2.22	3.13	2.73	0.36	101%	13%
ABO Pool 1:256	1.35	1.54	1.33	1.52	0.76	1.57	1.34	0.34	NC	25%
ABO Pool 1:512	0.67	0.87	-	-	0.51	0.95	0.77	0.23	NC	30%
ABO Pool 1:1024	0.34	-	-	-	-	-	NC	NC	NC	NC

The signal threshold was not reached   NC—Not calculated

Table S4. Characterization of limit of quantitation (LLOQ) of the anti-DENV3 complement-fixing antibody assay. Anti-DENV complement-fixing antibody assay was conducted againt all DENV serotypes. LLOQ analysis shown only include DENV3. Complement-fixing antibody concentrations against DENV3 were determined on five different occasions in one sample using multiple dilutions. Expected and averaged observed concentrations as well as percent (%) recovery and coefficient of variance (%CV; precision) were calculated. Rows highlighted in grey represent samples in which concentrations were calculated based on extrapolation of the titration curves (threshold signal was not reached) and, therefore, were considered not detectable.

Samples	Expected Concentration	Complement-Fixing Antibody Titer (EU/mL) DENV3 VLP (Obtained Concentration)								
		VBU-01140-184	VBU-01140-185	VBU-01140-188	VBU-01140-190	VBU-01140-193	Average	SD	% Recovery	%CV
Reference	369.00	-	-	-	-	-	-	-	-	-
ABO Pool 1:2	184.50	211.80	177.43	157.61	168.53	174.03	177.88	20.40	96%	11%
ABO Pool 1:4	92.25	104.33	94.66	93.56	80.86	76.36	89.96	11.28	98%	13%
ABO Pool 1:8	46.13	53.64	45.65	40.78	41.95	41.84	44.77	5.29	97%	12%
ABO Pool 1:16	23.06	23.68	22.19	20.25	22.14	20.53	21.76	1.40	94%	6%
ABO Pool 1:32	11.53	10.75	11.90	10.94	10.86	10.33	10.96	0.58	95%	5%
ABO Pool 1:64	5.77	6.05	5.41	6.30	4.70	5.96	5.68	0.64	99%	11%
ABO Pool 1:128	2.88	3.03	2.64	2.92	2.30	2.90	2.76	0.29	96%	11%
ABO Pool 1:256	1.44	1.21	1.12	1.20	0.63	1.02	1.04	0.24	NC	23%
ABO Pool 1:512	0.72	-	-	0.49	-	-	0.49	NC	NC	NC
ABO Pool 1:1024	0.36	-	-	-	-	-	NC	NC	NC	NC

The signal threshold was not reached NC—Not calculated

Table S5. Characterization of limit of quantitation (LLOQ) of the anti-DENV4 complement-fixing antibody assay. Anti-DENV complement-fixing antibody assay was conducted againt all DENV serotypes. LLOQ analysis shown only include DENV4. Complement-fixing antibody concentrations against DENV4 were determined on five different occasions in one sample using multiple dilutions. Expected and averaged observed concentrations as well as percent (%) recovery and coefficient of variance (%CV; precision) were calculated. Rows highlighted in grey represent samples in which concentrations were calculated based on extrapolation of the titration curves (threshold signal was not reached) and, therefore, were considered not detectable.

**Samples**	**Expected Concentration**	**Complement-fixing antibody Titer (EU/mL) DENV4 VLP (Obtained Concentration)**								
		**VBU-01140-184**	**VBU-01140-185**	**VBU-01140-188**	**VBU-01140-190**	**VBU-01140-193**	**Average**	**SD**	**% Recovery**	**%CV**
Reference	257.00	-	-	-	-	-	-	-	-	-
ABO Pool 1:2	128.50	151.94	122.52	101.84	108.87	121.68	121.37	19.19	94%	16%
ABO Pool 1:4	64.25	72.98	63.56	62.17	52.93	52.54	60.84	8.49	95%	14%
ABO Pool 1:8	32.13	37.13	30.94	26.45	27.23	27.58	29.86	4.41	93%	15%
ABO Pool 1:16	16.06	16.07	14.53	13.25	13.92	14.38	14.43	1.04	90%	7%
ABO Pool 1:32	8.03	7.04	8.04	7.25	6.90	7.12	7.27	0.45	91%	6%
ABO Pool 1:64	4.02	4.14	3.82	4.56	3.38	4.42	4.06	0.48	101%	12%
ABO Pool 1:128	2.01	2.14	1.86	2.15	1.68	2.17	2.00	0.22	100%	11%
ABO Pool 1:256	1.00	1.01	0.89	0.93	0.56	0.85	0.85	0.17	NC	20%
ABO Pool 1:512	0.50	0.53	-	0.43	-	0.39	0.45	0.08	NC	17%
ABO Pool 1:1024	0.25	-	-	-	-	-	NC	NC	NC	NC

The signal threshold was not reached

NC—Not calculated

Table S6. Summary of LLOQ of the anti-dengue virus complement-fixing antibody assay.

Dengue Serotype	LLOQ (EU/mL)
DENV1	4.0
DENV2	3.0
DENV3	3.0
DENV4	2.0

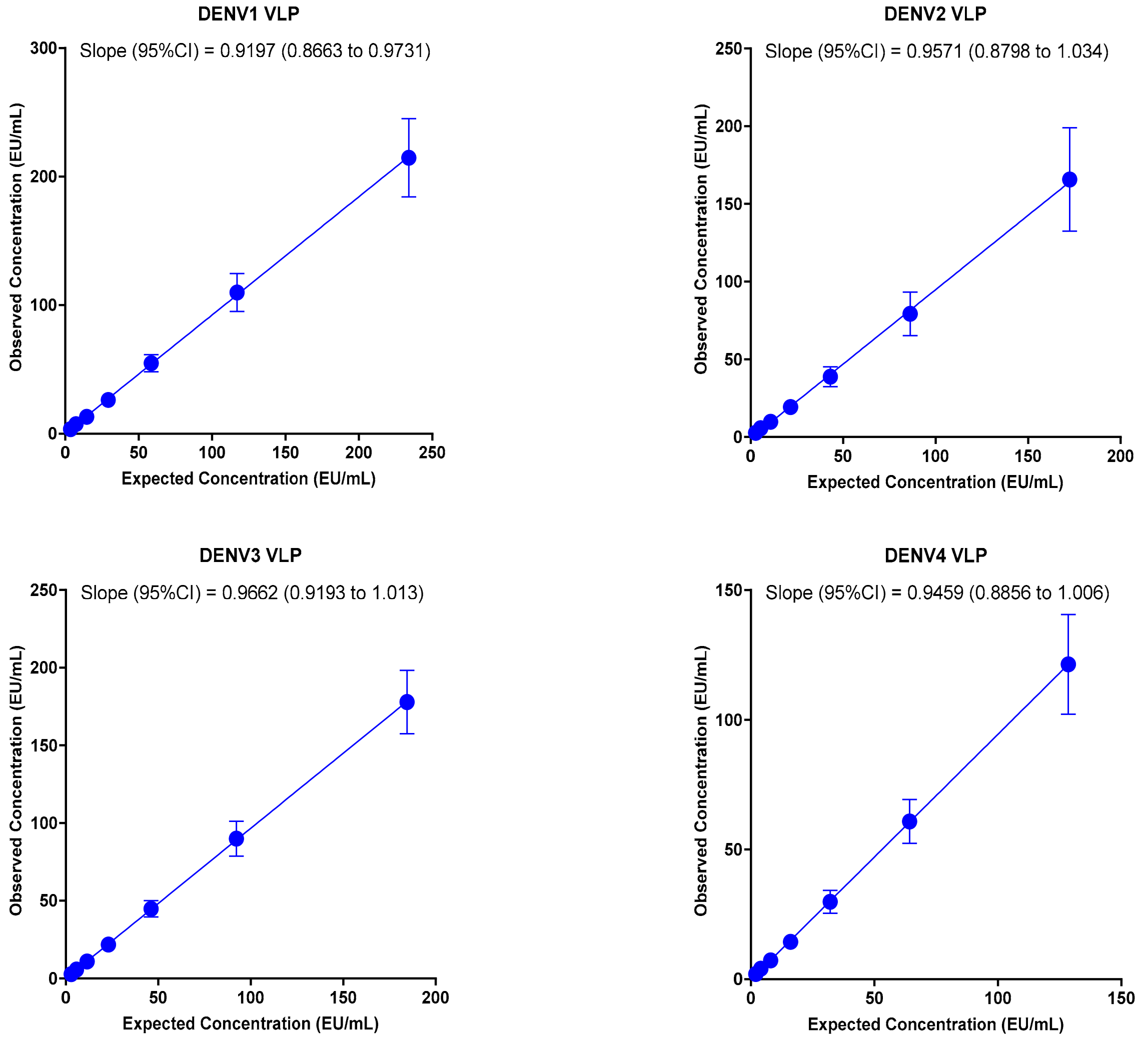

Figure S3. Linearity analysis of the anti-DENV complement-fixing antibody assay. Assay linearity was evaluated by plotting the value of the mean observed concentration of five independent measurements of a sample at different concentrations on the anti-DENV complement-fixing antibody assay. The slope of the linear regression of the data (95% confidence interval) was calculated for each of the DENV serotypes.

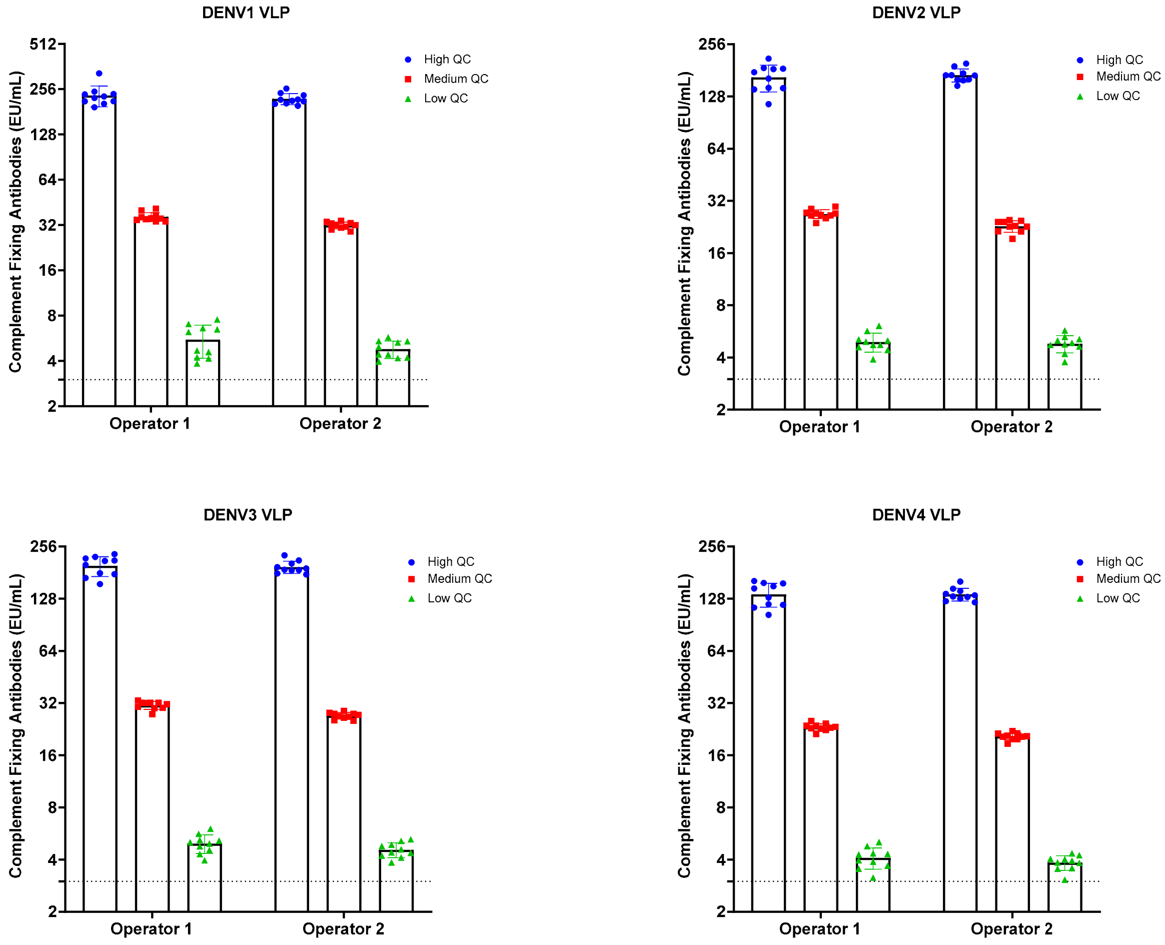

Figure S4. Anti-DENV complement-fixing antibody of assay controls measured by two operators on multiple days. Complement-fixing antibodies of the high, medium and low control samples were tested five times per run by two operators on two different occasions. Concentration of 3 EU/mL is shown as dotted line for reference.

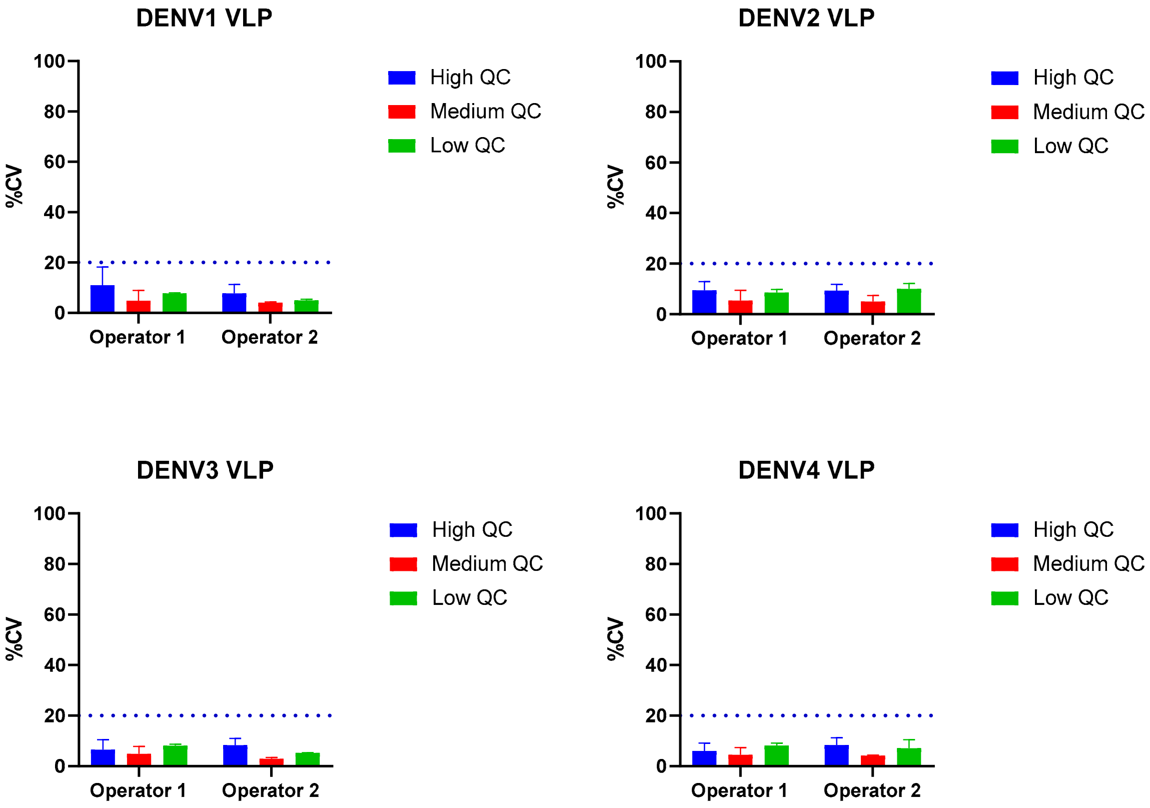

Figure S5. Reproducibility (per operator) of the Anti-DENV complement-fixing antibody assay. Coefficient of variance (%CV) measurement of anti-DENV complement-fixing antibody concentrations of the assay controls (high, medium and low) against each DENV serotype per operator. Dotted line represents 20% CV for reference.

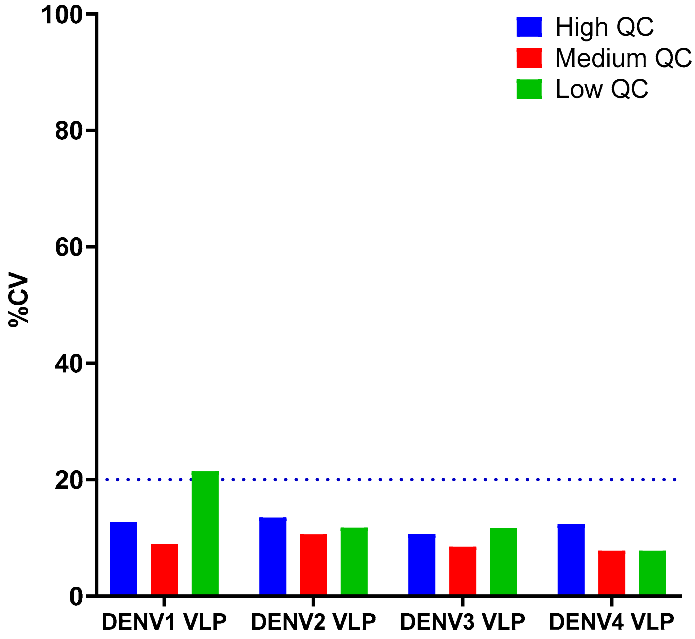

Figure S6. Reproducibility (all operators) of the Anti-DENV complement-fixing antibody assay. Coefficient of variance (%CV) measurement of anti-DENV complement-fixing antibodies of the assay controls (high, medium and low) against each DENV serotype considering both operators. Dotted line represents 20% CV for the reference.

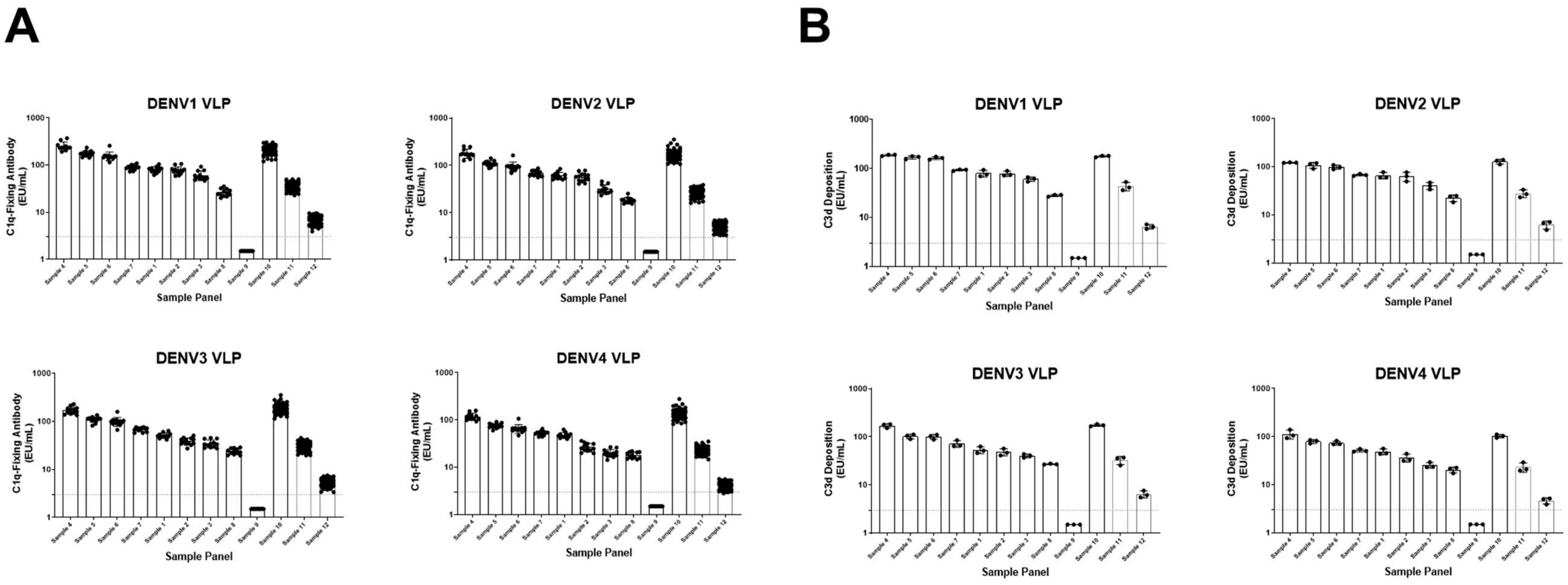

Figure S7. Complement-fixing antibody levels based on C1q fixation or C3d deposition. A panel of samples from healthy individuals from a dengue endemic area in Colombia were used to evaluate complement-fixing antibodies against each DENV serotype based on (**A**) C1q fixation or (**B**) C3d deposition. Concentration of 3 EU/mL is shown as dotted line for reference.

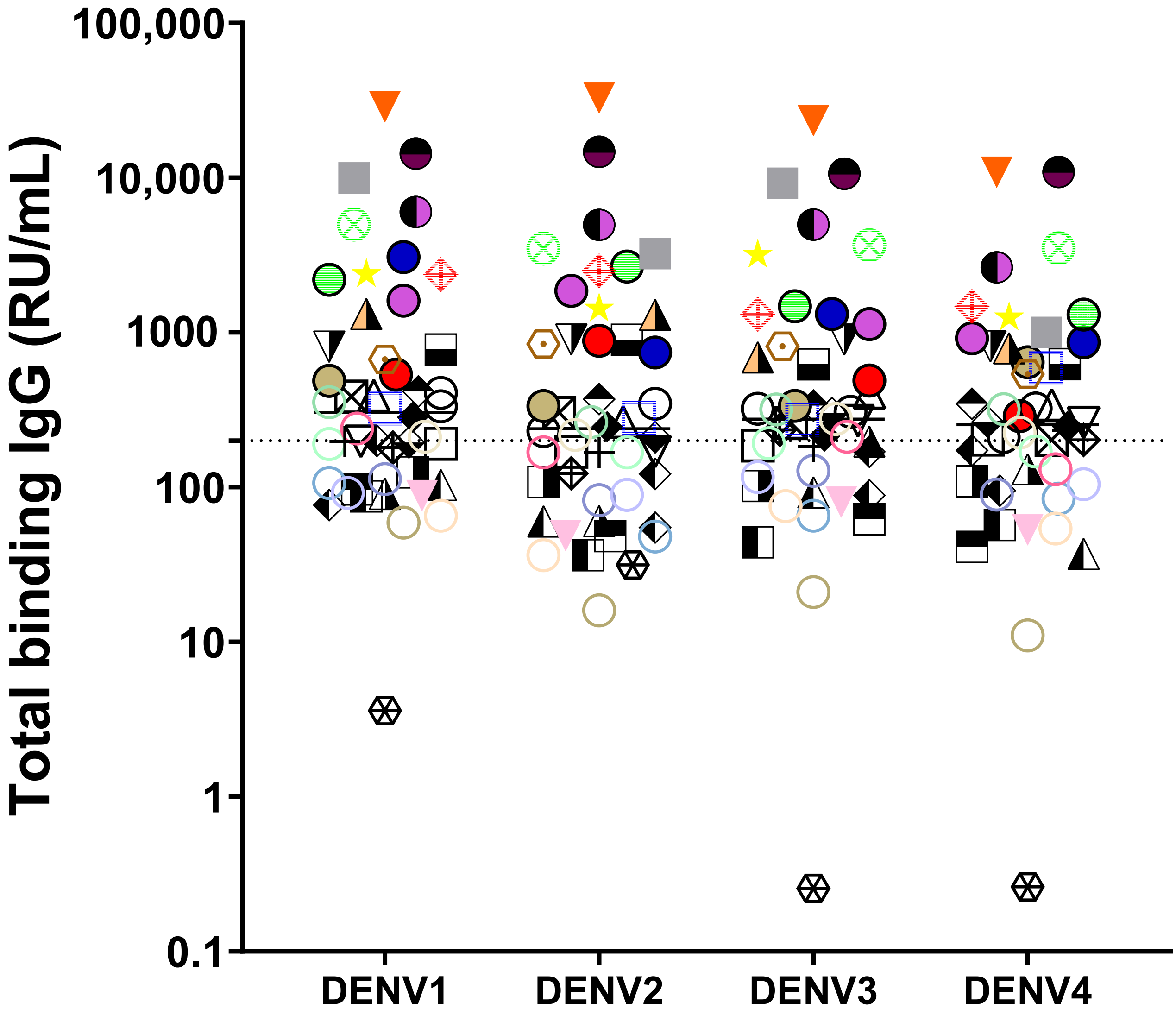

Figure S8. Total binding IgG concentration against all four dengue virus serotypes in subjects living in dengue endemic areas. Total binding IgG concentration was obtained from subjects participating in phase II clinical trials DEN-203 and DEN-204 before vaccination with tetravalent live-attenuated dengue vaccine TAK-003. Concentration of 200 RU/mL is shown as dotted line for reference.

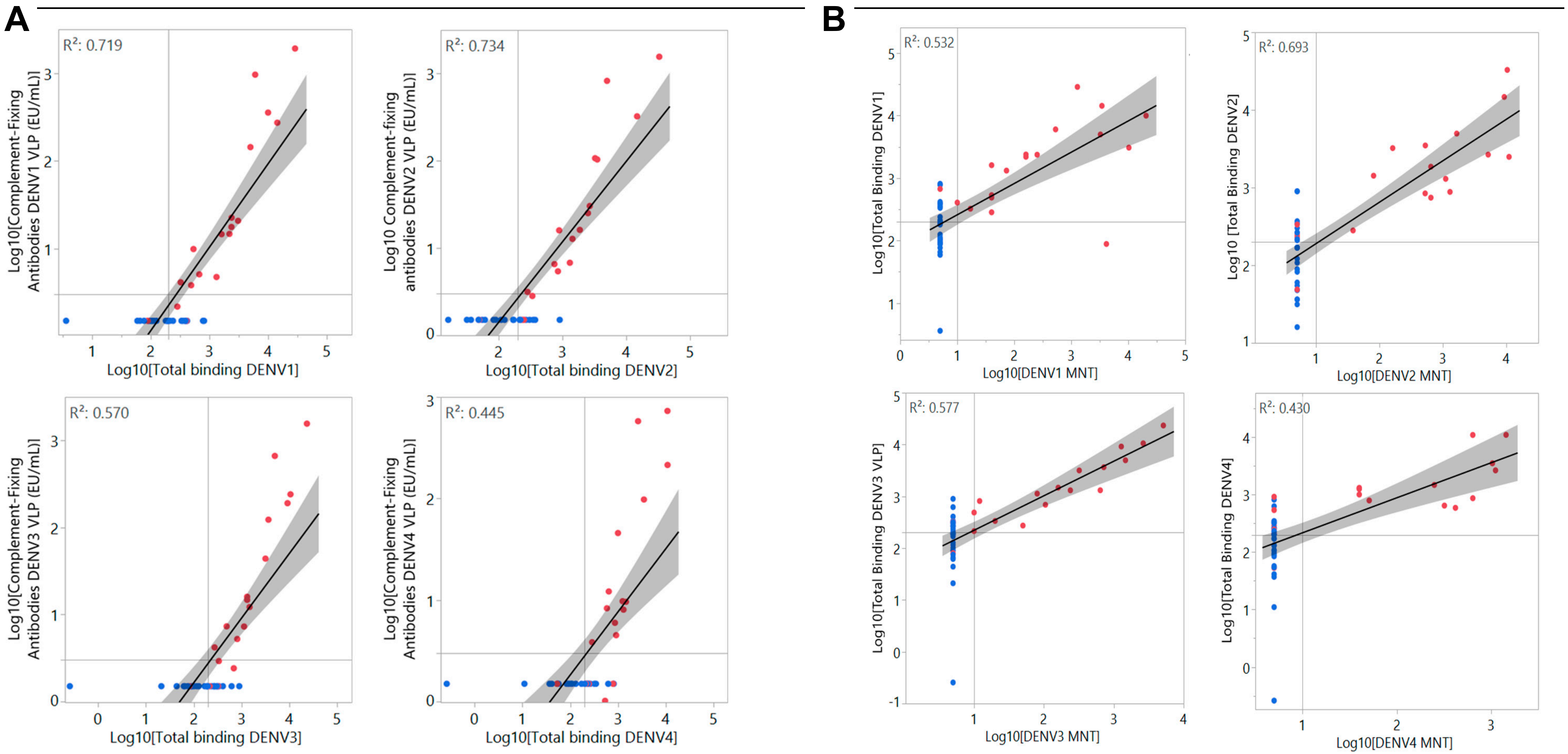

Figure S9. Correlation analysis between (**A**) total binding IgG concentration and complement-fixing antibodies or (**B**) MNT50 titers against all four dengue virus (DENV) serotypes in subjects living in dengue endemic areas. Correlation analysis was performed using Log_{10}-transformed total binding IgG and complement-fixing antibody concentrations or MNT50 titers and correlation coefficient (R^2) was calculated for each DENV serotype. Samples are color coded by DENV serostatus determined by MNT_{50}. Blue symbols represent seronegative study participants, and red represents seropositive ones. On (**A**) Vertical and horizontal lines represent total binding concentration of 200 RU/mL and complement-fixing antibody concentration of 3 EU/mL. On (**B**) Vertical and horizontal lines represent MNT50 titer of 10 and total binding concentration of 200 RU/mL. The shaded gray represents the confidence region for the fitted line.

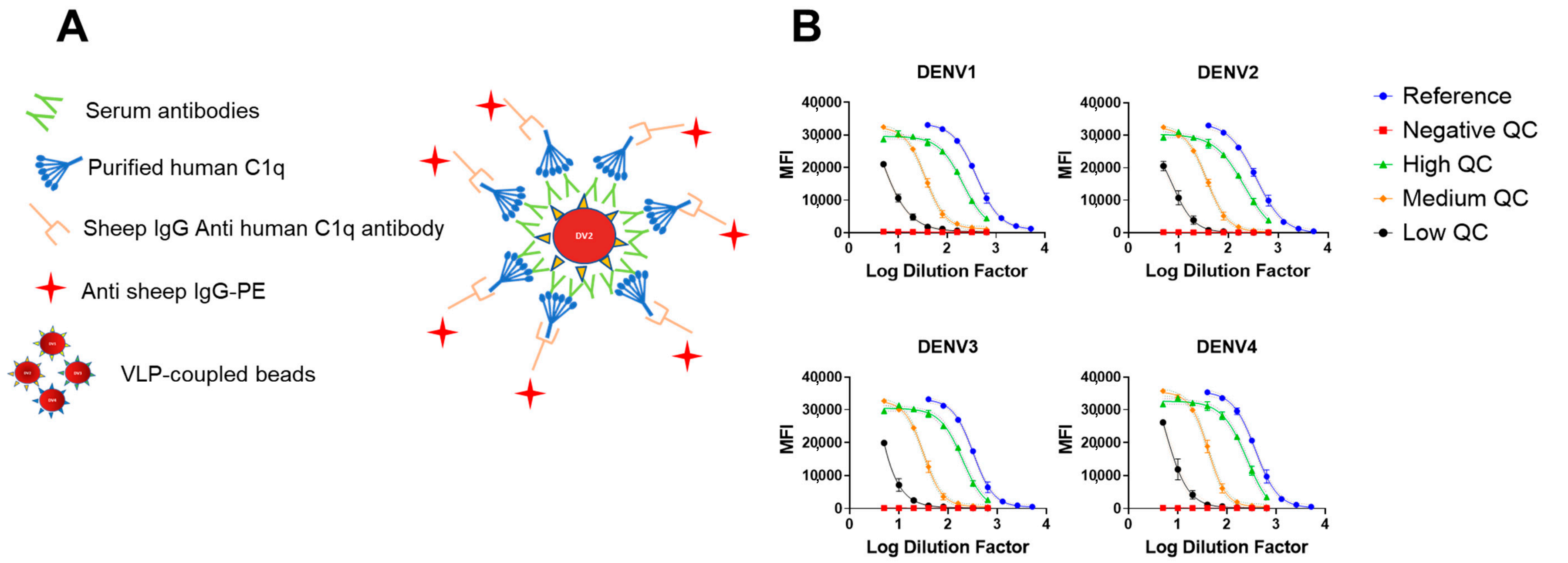

Figure S10. Anti-dengue virus (DENV) complement-fixing antibody assay design and representative titration curves. (**A**) Schematic overview of the anti-DENV complement-fixing antibody assay. The anti-DENV complement-fixing assay uses microspheres coupled to DENV1-4 virus-like particles (VLPs). The DENV VLP-coupled microspheres are applied as a mixture allowing detection of complement-fixing antibodies reactive to all of the different DENV serotypes in one single experiment. Samples evaluated in the anti-dengue virus complement-fixing antibody assay are heat inactivated at 56°C to inactivate endogenous C1q and other complement proteins. The VLP-coupled microspheres are incubated with a sample comprising complement-fixing antibodies to allow binding of these antibodies to the VLPs. Afterwards, purified human C1q is added to the mixture to bind complement-fixing antibodies from the sample bound to the VLPs. Bound human C1q is then further detected using an anti-human C1q pre-reporter antibody and an anti-sheep IgG phycoerythrin-coupled reporter antibody generating a fluorescence signal detected on the plate reader Magpix. (**B**) Titration curves of reference, positive (high, medium and low) and negative control samples on the anti-DENV complement-fixing antibody assay against each of the four DENV serotypes.
